# A Cohesin-Based Partitioning Mechanism Revealed upon Transcriptional Inactivation of Centromere

**DOI:** 10.1371/journal.pgen.1006021

**Published:** 2016-04-29

**Authors:** Michael Tsabar, Julian Haase, Benjamin Harrison, Chloe E. Snider, Brittany Eldridge, Lila Kaminsky, Rebecca M. Hine, James E. Haber, Kerry Bloom

**Affiliations:** 1 Department of Biology and Rosenstiel Basic Medical Sciences Research Center, Brandeis University, Waltham, Massachusetts, United States of America; 2 Department of Biology, University of North Carolina at Chapel Hill, Chapel Hill, North Carolina; Duke University, UNITED STATES

## Abstract

Transcriptional inactivation of the budding yeast centromere has been a widely used tool in studies of chromosome segregation and aneuploidy. In haploid cells when an essential chromosome contains a single conditionally inactivated centromere (*GAL-CEN*), cell growth rate is slowed and segregation fidelity is reduced; but colony formation is nearly 100%. Pedigree analysis revealed that only 30% of the time both mother and daughter cell inherit the *GAL-CEN* chromosome. The reduced segregation capacity of the *GAL-CEN* chromosome is further compromised upon reduction of pericentric cohesin (*mcm21∆*), as reflected in a further diminishment of the Mif2 kinetochore protein at *GAL-CEN*. By redistributing cohesin from the nucleolus to the pericentromere (by deleting *SIR2*), there is increased presence of the kinetochore protein Mif2 at *GAL-CEN* and restoration of cell viability. These studies identify the ability of cohesin to promote chromosome segregation via kinetochore assembly, in a situation where the centromere has been severely compromised.

## Author Summary

Studies of kinetochore organization and function led to the development of conditionally inactivated centromeres. The most commonly used conditionally inactivated centromere tool is the insertion of a galactose inducible promoter upstream of the centromeric sequence, termed *GAL-CEN*. Viability of haploid cells containing *GAL-CEN3* grown on galactose is close to 100%, despite having an inactivated centromere. Inactivation of *CEN3* leads to aberrant segregation of sister chromatids in metaphase, and an impairment in recruitment of centromeric proteins. Strikingly, when pericentromeric cohesin recruitment is impaired by deleting *MCM21*, viability is reduced to 23%. Moreover, *mcm21Δ GAL-CEN3* cells demonstrate a more pronounced sister chromatid segregation defect and reduced recruitment of the kinetochore protein Mif2 as compared to GAL-CEN3 alone. The defects observed in *mcm21Δ* are rescued to *GAL-CEN3* WT levels by deletion of *SIR2*, which restores cohesin recruitment to the pericentromeric regions in *mcm21Δ*. Our data suggests cohesin plays a role in centromere function to serve as a template for proper kinetochore structure.

## Introduction

Proper microtubule attachment is required for accurate chromosome segregation. Attachment to the mitotic spindle requires the formation of a multiprotein kinetochore at the specialized chromosomal locus, the centromere. Studies of how kinetochores are specified led to the development of conditionally functional centromeres [1,2]. The most common of these makes use of a galactose-inducible promoter placed upstream from the centromeric DNA sequence. Termed *GAL-CEN*, this conditional centromere is functional when cells are grown on glucose but its function is inhibited when cells are grown on galactose [2]. Both chromosomes and autonomous mini-chromosomes harboring the *GAL-CEN3* construct show severe defects in chromosome segregation upon kinetochore inactivation on galactose. In haploid cells carrying a nonessential *GAL-CEN3* plasmid, or diploids carrying a single *GAL-CEN3* chromosome, the percentage of *GAL-CEN3* containing cells dropped to less than 5–10% within 10 generations following centromere inactivation [2]. Subsequent studies found that cells containing *GAL-CEN3* plasmids showed a biased segregation pattern with low copy plasmids accumulating in the mother cell (~8 copies after 3 divisions, [3]). The transcriptional inactivation of a centromere has been widely used to study consequences of aneuploidy [4,5,6].

The mechanism of transcriptional inactivation has not been established. Chlebowicz-Sledzieswka *et al*. [1] were able to detect RNA transcripts through the centromere. However, using micrococcal nuclease to probe the region of centromere-binding proteins, Hill and Bloom [2] found that the area of protection against nuclease digestion was indistinguishable from the wild-type active centromere. Transcriptional inactivation is not mediated by the complete removal of kinetochore proteins. Collins *et al*., [7] using chromatin immunoprecipitation (ChIP), found that a suite of kinetochore proteins remain bound to the DNA, on average at 10–30% the levels of wild-type active centromeres. These studies indicate that kinetochore proteins are perturbed or removed in a fraction of cells, drastically reducing the segregation capabilities of the centromere.

Cohesin and condensin protein complexes are enriched 3-fold in the region surrounding the centromeres relative to the bulk chromosome arms [8,9,10,11,12]. Cohesin does not promote sister centromere cohesion per se, as centromeric sister chromatids under tension are well-separated relative to sites on chromosome arms [13]. Instead, cohesin has been shown to stabilize intramolecular loops in the pericentromere (3C, chromosome conformation capture) [14]. Transcriptional inactivation of the centromere disrupts the ability of the pericentromere to adopt the loop conformation, as the disruption of centromere via juxtaposition to an active promoter alters the conformation of the entire 50 kb pericentromere loop [15]. Thus the higher order conformation is dependent on local interactions.

This study utilizes the conditional *GAL-CEN3* as the only chromosome 3 (Chr 3) centromere in a haploid cell. Cells with *GAL-CEN3* are able to form colonies efficiently on galactose, even though the centromere has been “inactivated”. Reduction of pericentric cohesin in a *mcm21*Δ mutant, but not reducing condensin, markedly reduces *GAL-CEN3* colony formation. Recruiting cohesin to the pericentromere by preventing its Sir2-dependent assembly in ribosomal DNA (rDNA) suppresses *mcm21*Δ. We demonstrate that these changes in viability are reflected in the concentration of the kinetochore protein Mif2 at *CEN3* and in the level of sister centromere separation established in metaphase. Thus, centromeric cohesin contributes to mechanisms of chromosome segregation for chromosomes with transcriptionally compromised centromeres.

## Results

### Viability of haploid *GAL-CEN3*-containing cells

**Fig 1:**
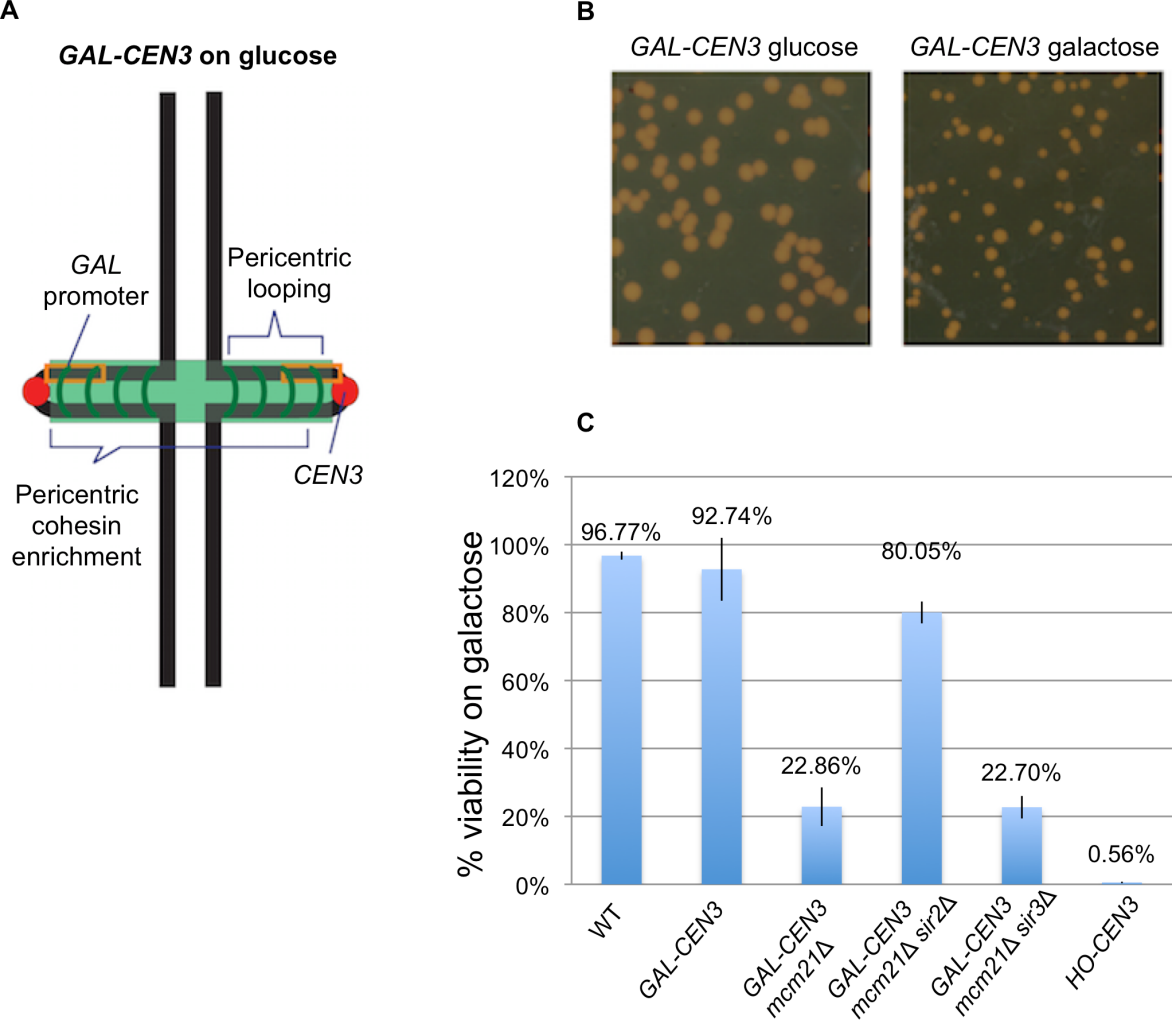
Viability of cells containing the *GAL-CEN3* chromosome. A. Schematic of the *GAL-CEN3* chromosome. The *GAL-CEN3* chromosome contains a *GAL1* promoter (orange box) adjacent to *CEN3* on Chr 3 [2]. A LacO array was integrated approximately 3.8 kilobases from the centromere sequence of the *GAL-CEN3* construct. The centroid of the LacO array is 8.8 kb from *CEN3*. Thick black lines represent chromosome arms. The chromosome is drawn based upon direct observations in live cells. The centromeres (red) are separated by approximately 800 nm. Cohesin (green) is enriched in the pericentromere region, about 50 kb surrounding each centromere. B. Representative cells grown on glucose or galactose. Glucose plates shown were imaged at 48 hours, galactose plates at 120 hours. C. Viability was derived from the percentage of colony forming units on galactose versus glucose. Wild type cells are the background strain not containing the *GAL-CEN3* chromosome. From the left are *GAL-CEN3*, *GAL-CEN3 mcm21Δ*, *GAL-CEN3 mcm21Δ sir2Δ*, *GAL-CEN3 mcm21Δ sir3Δ* and HO-*CEN3*.

To assess the viability of a haploid strain containing a *GAL-CEN3* chromosome, we replaced *CEN3* with a *GAL-CEN3* and measured cellular viability on glucose- and galactose-containing media (Fig 1). *GAL-CEN3* is contained on an 865 bp fragment that does not include the *GAL10* transcription initiation site [2] and thus transcription is only directed toward *CEN3*. *GAL-CEN3* containing haploid cells formed colonies after 120 h of incubation on galactose (Fig 1). These colonies were comparable in size to those of wild type cells incubated for 48 h (Fig 1). Despite the slow growth defect, these cells showed ~92% colony-forming ability on galactose suggesting that the *GAL-CEN3* containing chromosome is not fully lost from the population and that sister *CEN3*s segregate to daughter cells often enough to generate a colony (Fig 1).

### Pedigree analysis of cells containing *GAL-CEN* chromosomes

**Fig 2:**
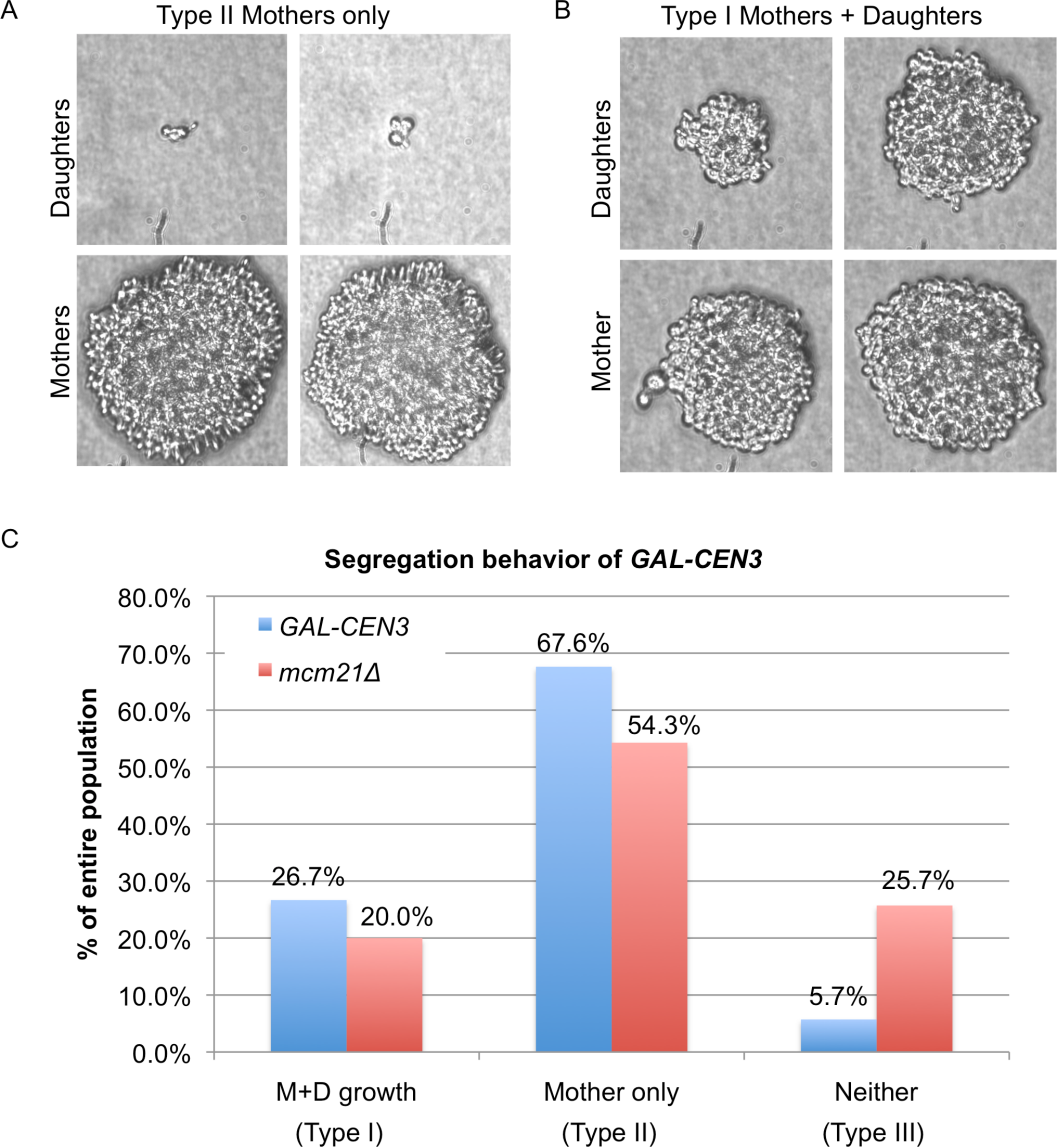
Pedigree analysis of *GAL-CEN* chromosome distribution. Individual G1 cells were micromanipulated into an array on a YEP galactose-containing plate and were monitored microscopically. When a cell had completed budding and a new bud just appeared one of the cells (the slightly larger, mother cell), the mother and daughter cells were separated by micromanipulation and then observed approximately 24 hrs later to determine if the cell had grown into a microcolony of >20 cells or had arrested either as a single dumbbell or as a microcolony of <8 cells. Despite the presence of several essential genes on Chr 3, cells are able to divide at least once without transcription. Images of microcolonies were photographed. A. Only mother cells (Type II) divided multiple times. B. Both mother and daughter (Type I) cells divided multiple times. C. Quantification of progeny analysis. For *GAL-CEN3* wild-type (blue) n = 105 and *GAL-CEN3 mcm21*∆ (red) n = 70. Type I: Mother viable, Daughter viable; Type II: Mother viable, Daughter dead; Type III: neither cell viable.

To investigate the ability of *GAL-CEN3* containing cells to form colonies, we carried out a detailed pedigree analysis of chromosome transmission. Cells grown on glucose medium were plated on galactose-containing agar. Unbudded G1 cells were followed under the microscope and mother cells (which are larger and initiate a new bud earlier than their daughters on rich medium) were separated from their daughters. These cells were then observed as they continued to grow and divide. For *GAL-CEN3* on Chr 3, in approximately 26.7% of the mother/daughter pairs (28/105) both mother and daughter cells continued to divide with no apparent delay over a period of 12 h, producing two microcolonies (M and D growth Type I, Fig 2). In most of the remaining cells (67.6% 71/105), the mother cell grew into a colony while the daughter produced cells that apparently failed to divide and arrested as enlarged dumbbells or else divided once or twice to produce inviable microcolonies (Mother only, Type II). We conclude that these cases represent the failure to transmit Chr 3 to the daughter. In six cases, neither mother nor daughter cell grew into a colony (Fig 2 Type III). Further analysis of Type I segregants showed that inviable cells were generated in later cell divisions, but these were less frequent in the mother cells of Type II segregants, where presumably the mother now carried two copies of *GAL-CEN3*, each of which could apparently behave independently.

We extended this study to examine the segregation behavior of a set of 4 haploid strains generated by Reid *et al*., [6] in which each strain contains a different *GAL-CEN* chromosome. Overall, the results for *GAL-CENs* in chromosomes 2, 3, 4 and 5, were comparable as described for Chr 3 above, in which 25–55% of cells gave rise to viable mother and daughter cells (Type I) and 45–58% of cells gave rise to viable mother cells only (Type II, S1 Fig). The frequency of no viable growth was between 10 and 15% (S1 Fig). The variability in pedigree may reflect differences from the precise positioning of the *GAL1* enhancer/promoter relative to the *CEN* sequences (Hill and Bloom [2], *GAL-CEN3* Fig 2 vs. Reid *et al*., [6], *GAL-CEN3* S1 Fig).

We note that some daughter cells that had apparently lost a chromosome could nevertheless divide one or more times before arresting, despite the fact that every chromosome has at least one cell division cycle (*CDC*) gene whose action is required in every cell division [16,17]. For example, in daughters that appeared to have lost Chr 3 at the first division, most of the microcolonies had 4–6 cells. We suggest that in some instances the gene product is not turned over every cell division and thus can persist for one or more generations even when the template for further transcription is absent. Consistent with this idea, daughters losing Chr 4, which contains *CDC20*, whose product is destroyed every cell cycle, only produce dumbbell-shaped daughters. In a few instances we also observed that mother colonies had unusual cell morphologies, which may reflect the over expression of genes on the mis-segregated chromosome, consistent with other studies of aneuploidy in yeast [18].

### *GAL-CEN3* chromosome behavior

**Fig 3:**
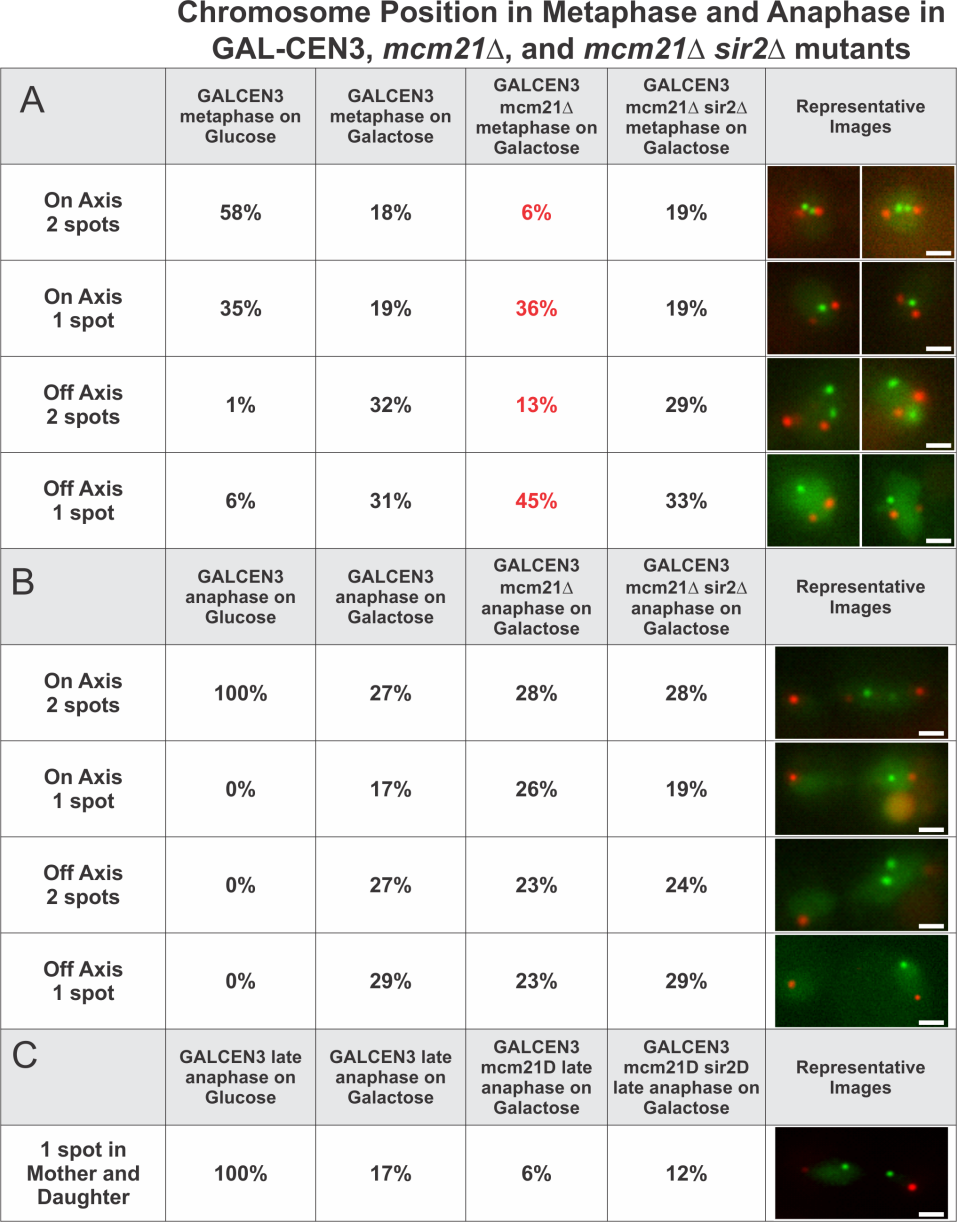
Centromere-linked LacO position in wildtype and anaphase cells. GAL-CEN3 proximal lacO arrays in metaphase (A) (n = 84 to 92 cells) and anaphase (B) (n = 34 to 51 cells) cells. Cells were grown on galactose for 3h prior to image analysis. The fraction of replicated spots that appeared as one or two foci between the spindle poles and along the spindle axis (on axis), versus the fraction of replicated spots that appeared as one or two foci displaced from the spindle axis was determined. Representative images are shown to the right. In wildtype cells with endogenous CEN3 as the sole centromere in Chr 3, the lacO array appeared as a single focus (30%) or separated foci (70%) on the spindle axis of metaphase cells. In mcm21Δ cells with GAL-CEN3 as the sole centromere in Chr 3, the distribution of GAL-CEN3 lacO arrays was comparable to cells with GAL-CEN3 on glucose (metaphase 2 spots on axis 62%, one spot on axis 30%, two spots off axis 2%, one spot off axis 7%; anaphase on axis 2 spots 100%, (metaphase n = 60, anaphase n = 22). In mcm21Δ sir2Δ cells with GAL-CEN3 as the sole centromere in Chr 3, the distribution of GAL-CEN3 lacO arrays was comparable to cells with GAL-CEN3 on glucose (metaphase 2 spots on axis 57%, one spot on axis 34%, two spots off axis 1%, one spot off axis 7%; anaphase on axis 2 spots 100%, (metaphase n = 82, anaphase n = 25). The fraction of cells with two spots in late anaphase is shown (bottom panel). In wild type cells with endogenous CEN3 as the sole centromere in Chr 3, the lacO array appeared as two foci, one at each pole of the anaphase spindle in 100% of cells. In mcm21Δ cells with GAL-CEN3, 100% of spots in late anaphase appeared in mother and daughter cells on glucose (n = 22). In mcm21Δ sir2Δ cells with GAL-CEN3, 100% of spots in late anaphase appeared in mother and daughter cells on glucose (n = 25). Panel C indicates the percent of cells from each sample in which lacO arrays segregate to mother and daughter in late anaphase. 17 and 12% of GAL-CEN3 WT (n = 18 cells) and mcm2∆ sir2∆ mutants (n = 26 cells) contain two foci segregated to mother and daughter, while only 5% of mcm21∆ cells (n = 18 cells) exhibit this phenotype.

When replicated chromosomes are properly attached to kinetochore microtubules emanating from opposite spindle pole bodies, tension across sister centromeres results in their physical separation in metaphase. Using a LacO array integrated 8.8 kb (centroid of the LacO array) from the centromere and expressing LacI-GFP, sister LacO arrays in the pericentromeric region appear as either two distinct spots or a single focus depending on the distance between replicated sister chromatids [19]. When grown on glucose, separated sister LacO arrays were observed in 59% (58% on-axis + 1% off-axis) of cells containing a metaphase length spindle (1.5–2.0 μm, tracked using the spindle pole protein SPC29 fused to RFP; Fig 3). In the remaining 41% (35% on-axis + 6% off-axis), the sister LacO arrays formed a single focus. Whether they appeared as one spot or two, in wild type cells sister LacO arrays reside between the spindle poles and within 200 nm from the spindle axis in greater than 90% of metaphase cells [20].

Changes in metaphase centromere alignment are observed after shifting an asynchronous population to galactose for 3 h (*GAL-CEN3* metaphase, Fig 3). The most prominent phenotypes for sister centromeres were: two foci off the spindle axis (32%), one focus off the spindle axis (31%), two foci on the spindle axis (18%) or one focus on the spindle axis (19%) (Fig 3). If the *GAL-CEN3* centromere were completely non-functional, the prediction is that sister foci would rarely be separated as characteristic of non-centromeric chromosome arms. The large fraction of cells with off axis foci (62%) indicate the loss of centromere function, consistent with the finding that about 50% of *GAL-CEN3* centromeres are inactivated in the first cell cycle upon transfer to galactose [4]. Separated sister LacO arrays on the spindle axis were apparent in 18% of cells, suggesting proper biorientation and tension in a fraction of *GAL-CEN3*-containing chromosomes. Alternatively, it is possible that the spindle-proximal foci are not attached to kinetochore microtubules, but the sister centromeres are separated independent of microtubules. In any case, the proportion of cells that partitioned *GAL-CEN3* (~50%) was much greater than the 18% displaying apparently proper biorientation.

### *GAL-CEN3* chromosome partitioning in anaphase

Based upon chromosome position and colony growth on galactose, we hypothesized that roughly 50% of cells must partition the *GAL-CEN3* chromosome during cell division. As a cell progresses from metaphase to anaphase, the spindle will transition from its metaphase length of 1.2–2.0 μm to anaphase lengths of 7–10 μm with sister centromeres moving apart [19,21]. In wild-type cells, 100% of cells contain a LacO focus associated at each spindle pole in anaphase (Fig 3). Inactivation of the *GAL-CEN3* chromosome after 3 h growth on galactose gave rise to multiple phenotypes in anaphase (spindles > 2 μm). About 46% (17% on-axis +29% off-axis) of cells had a single focus in the mother cell either on or off the spindle axis and 54% (27% on-axis +27% off-axis) of cells had two foci in the mother cell (Fig 3). In 13.5% of cells mother and daughter spindle poles each had a focus (~½ of the On Axis 2 spots, Fig 3). This is concordant with the fraction of successfully segregated sisters chromatids observed in anaphase (17%, 1 spot in mother and daughter, panel C, Fig 3). The 17% of anaphase segregation is less than the fraction (27%) of both mother and daughter cell receiving the *GAL-CEN3* chromosome in the first division in the pedigree analysis (Fig 2). The difference may reflect the physical consequences of micromanipulation (pedigree analysis) vs. exposure to high intensity light (live cell analysis). In the other half of cells with two foci aligned on the axis in anaphase, the foci lagged relative to the spindle poles and were often found in the mother cell, near the neck of the budded cell (shown in representative images to right, Fig 3). Rarely were foci observed only in the daughter bud (7% as one focus, 6% as two foci). Thus, in galactose-treated cells the *GAL-CEN3* chromosome can be segregated in a timely fashion, but more often lags behind wild type chromosome segregation.

**Fig 4:**
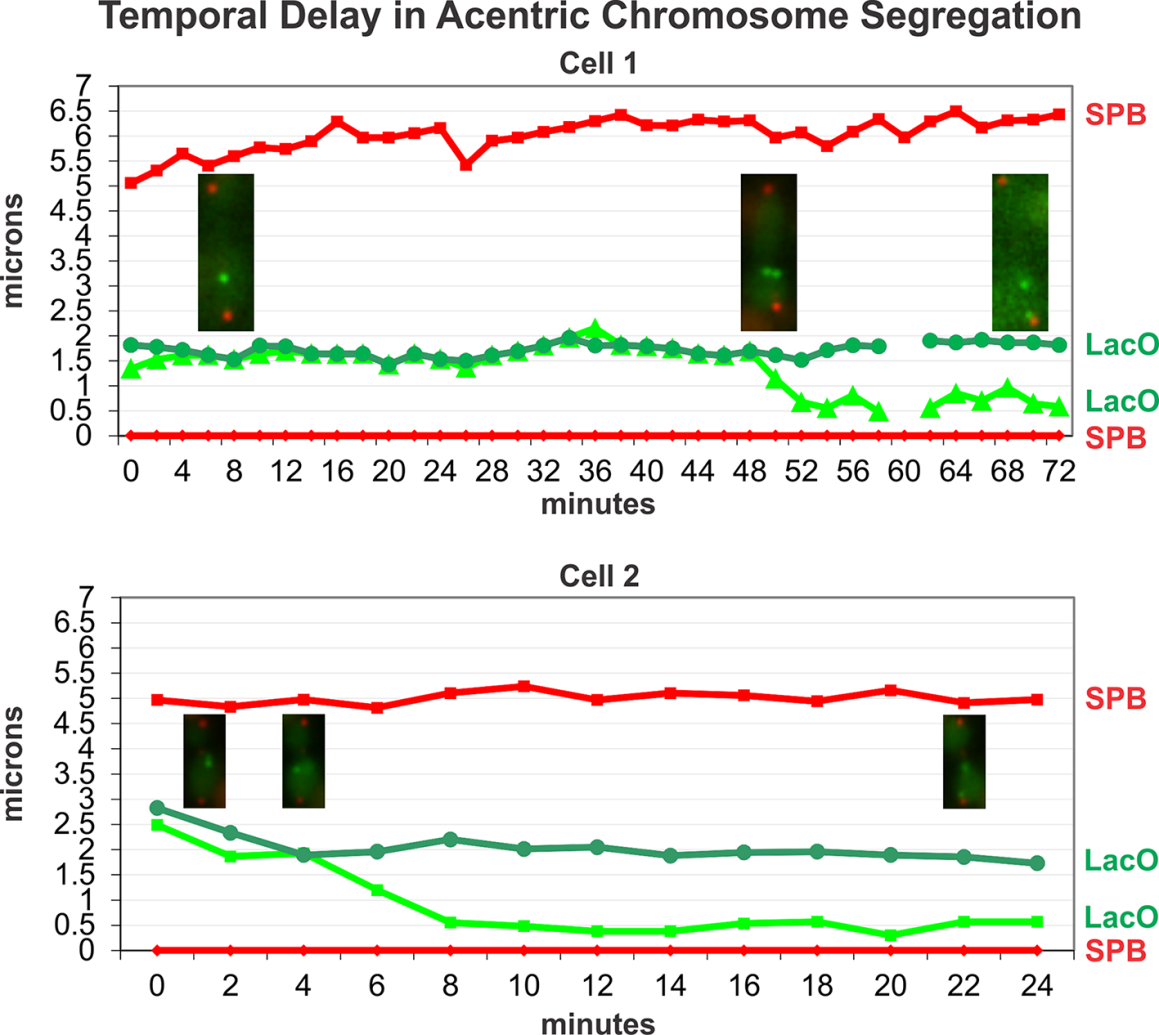
Poleward movement in cells grown on galactose. Cells with elongated anaphase spindles and *GAL-CEN3* proximal LacO arrays were imaged over time. Distances (microns) were measured in reference to a single spindle pole (indicated at position 0 micron over all time points). Pole to pole distance is depicted as the position from reference pole (red diamonds) to the second spindle pole (red squares), pole to each LacO focus is depicted in shades of green. LacO focus to spindle pole movement was observed for one of the LacO foci in each time lapse. The rate of movement is 0.29 μm/min ± 0.06, approx. 1/3 the rate of wild-type chromosome to pole movement. The distance traveled is 1.18 μm ± 0.23. Traces from 2 individual cells are shown (top and bottom). Time 0 is an arbitrary point in anaphase. Anaphase onset occurs at approx. 2 μm spindle length at a rate of ~ 1 μm/min. Endogenous centromeres move to the spindle pole coincident with anaphase onset [19]. Traces from individual cells highlight the finding that separation of *GAL-CEN3* is significantly delayed relative to anaphase onset and separation of endogenous sister centromeres. Inset: Images of cells at representative time points. Red spindle poles Spc29-RFP, indicated in squares and diamonds; Green LacI-GFP, LacO array integrated 8.8 kb (centroid) from *CEN3*. Sister foci are indicated in green circles and triangles (Cell 1), circles and small squares (Cell 2).

To further investigate the observation of lagging chromosome segregation, LacO arrays linked to *GAL-CEN3* were tracked using time-lapse microscopy following growth on galactose for 3 hours. The LacO focus exhibited poleward motion at a rate of 0.29 ± 0.06 μm/min over an average period of ~4 minutes (Fig 4). The LacO array traveled 1.18 ± 0.23 μm, bringing it to about 0.5 μm from the spindle pole. These centromere-linked foci moved to the pole later than wild-type centromeres whose movements to the pole coincided with anaphase onset. However, the rate of movement of *GAL-CEN3* linked foci was only about 1/3 the rate of endogenous centromere segregation (1 μm/min).

### Acentric chromosome behavior

The low incidence of segregation could result from the transient activation of the *GAL-CEN3* centromere, or a novel, albeit inefficient segregation mechanism. To completely remove the centromere, we introduced an HO cut site adjacent to *CEN3* and flanked the centromere with identical 2-kb DNA sequence so that 5’ to 3’ resection and repair of the DSB by single-strand annealing (SSA) leads to a complete deletion of *CEN3* [22] (S2 Fig). Galactose-mediated induction of HO endonuclease cleavage is essentially 100% efficient, and by 3 h, nearly all cells have deleted *CEN3* [22]. Upon *CEN3* excision, colony formation is completely abrogated on galactose, to < 0.5% viability (Figs 1 and S2). The ability of cells containing transcriptionally inactivated centromeres to segregate chromosomes to the daughter cell is therefore centromere-dependent. Fluctuations in transcription may allow transient centromere function, sufficient for irregular or slowed cell divisions and colony formation.

### Pericentric cohesin contributes to sister centromere separation

**Fig 5:**
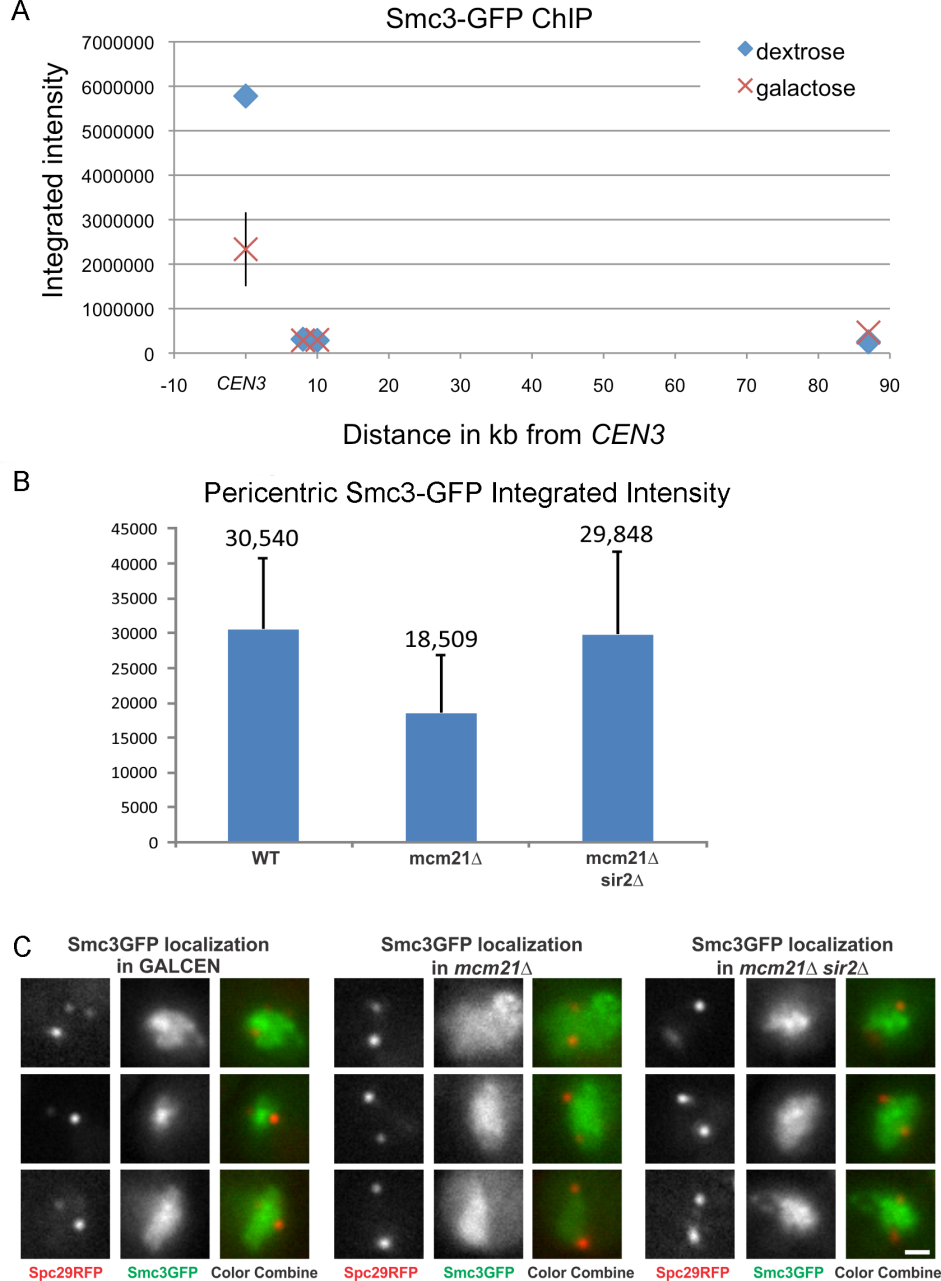
Cohesin concentration in the pericentric region in *GAL-CEN3*, *mcm21Δ* and *mcm21Δ*, *sir2Δ* mutants. A. Cells containing *GAL-CEN3* as the only centromere in chromosome 3 were transferred from lactose to galactose and grown on galactose for over 6 hours to inactivate the centromere. Chromatin immunoprecipitation was performed as described in Snider et al., (2014) [27], using a ChIP grade antibody against GFP to immunoprecipitate the only copy of Smc3 fused to GFP at the C-terminus of Smc3 in the genome. Oligonucleotide primers against *CEN3* (114,800), 8 kb (Stp22, position 105696..106853), 10 kb (Ilv6, position 104619..105548) and 87 kb (kar4 27929..28936) were utilized. Primers were designed to amplify a 600 bp fragment for each of the 4 reactions. Titrations of template were performed to ensure the analysis was in the linear range of amplification. For *GAL-CEN3* glucose, the integrated intensity ranged from 5.5 x 10^6 to 6.1 x 10^6 (indicated by glucose text inset in graph)(n = 4). The average was 5.78 x 10^6 ± 2.5 x 10^5 (STD). For GAL-CEN3 galactose, integrated intensity ranged from 1.2 x 10^6 to 3.3 x 10^6 (indicated by galactose text inset in graph)(n = 4). The average was 2.33 x 10^6 ± 8.3 x 10^5 (STD). For *STP22* (8 kb), *ILV6* (10 kb) and *KAR4* (87 kb) the galactose and glucose products ranged from 2.4 x 10^5 to 5.6 x 10^5 (n = 3). There was no significant difference between glucose and galactose grown samples for the 8, 10 or 87 kb products. B. The concentration of Smc3-GFP was determined in *GAL-CEN3* WT, *mcm21*∆ and *mcm21*∆ *sir2*∆ mutants. The concentration of pericentric cohesin is reduced in *mcm21*∆ cells (from 30,540 to 18,509 arbitrary fluorescence units). In the double mutant, *mcm21*∆ *sir2*∆, the concentration of cohesin in the pericentromere is increased to almost wild-type levels (29,848 vs 30,540). C. Representative images of Smc3-GFP in *GAL-CEN3* WT (left), *mcm21*∆ (middle) and *mcm21*∆ *sir2*∆ (right). Spindle poles are visualized using Spc29-RFP, cohesin with Smc3-GFP. The rightmost image in each strain is an overlay of the spindle poles with Smc3-GFP. White arrows indicate the cohesin barrel concentrated in the pericentric chromatin between the spindle poles (red). Note the absence of the cohesin barrel in *mcm21Δ*.

Cohesin plays an important structural role in chromosome bi-orientation, sister chromatid cohesion and 2 μ plasmid segregation [23,24,25]. Loading of cohesin at the centromere is mediated by the COMA complex [26]. Cohesin is 3-fold enriched in the pericentromere relative to chromosome arms. Upon transcriptional inactivation of the centromere by growth on galactose, cohesin levels (Smc3-GFP) are reduced to about 40% of the levels of wild-type (Fig 5A). The concentration of cohesin along chromosome arms was not sensitive to carbon source (Fig 5A). To test the role of pericentric cohesin in *GAL-CEN3* chromosome segregation we depleted cohesin from the pericentric chromatin via removal of Mcm21. Mcm21 (of the COMA complex) is a non-essential kinetochore component responsible for the enrichment of cohesin in the pericentromere [26]. The viability of *mcm21*∆ cells containing the *GAL-CEN3* chromosome was significantly reduced on galactose (22% *mcm21∆* vs. 92% *GAL-CEN3* WT, Fig 1). Like their wild-type counterparts, *mcm21*∆ cells in which the centromere was excised were largely inviable (<1.0%) (S2 Fig). The increased concentration of cohesin within the pericentromere may create barriers that prevent transcription and allow transient kinetochore function of the *GAL-CEN3* chromosome. The effect is specific for cohesin as 93% of *GAL-CEN3* cells with a 60% reduction in pericentric condensin (*cbf5-AUU*) [27] were viable (n> 1300 cells, 3 independent trials). *cbf5-AUU* is a nonessential mutation in the first AUG codon to AUU previously shown to alleviate repression by tRNA genes (*art1-1*; [28]) and reduce pericentric condensin [27].

To understand how pericentric cohesin might bias a transcriptionally inactive centromere toward the active state, we examined the localization of the *GAL-CEN3* chromosome in *mcm21∆* mutants (Fig 3). In wild-type cells, 59% (58% on-axis +1% off-axis) of the LacO spots located 8.8 kb from the centromere appear as separated spots. In contrast only ~20% (6% on-axis + 13% off-axis, Fig 3) of the LacO spots on the *GAL-CEN3* chromosome were separated in metaphase in the absence of *mcm21∆*. The ability for centromere-linked LacO arrays to separate or remain separated is significantly impaired upon reduction of pericentric cohesin in metaphase from logarithmically growing cell. This contrasts the role of cohesin throughout chromosome arms, where loss of cohesin via *mcm21Δ* results in *increased* sister separation (S3 Fig; LacO at 240kb 30% 2 spots in WT versus 47% in *mcm21Δ*). Pericentric cohesin thus contributes to sister centromere separation, not cohesion between sister centromeres.

In late anaphase, while there is a similar distribution of separated spots in wild type and *mcm21*∆ mutants (Fig 3C), only 6% of the LacO spots separate into mother and daughter in *mcm21*∆ vs. 17% in *GAL-CEN3* chromosome containing wild type cells (Fig 3C). The ~3-fold reduction in mother-daughter partitioning coincides with the ~4-fold reduction in colony viability (Fig 1).

**Fig 6:**
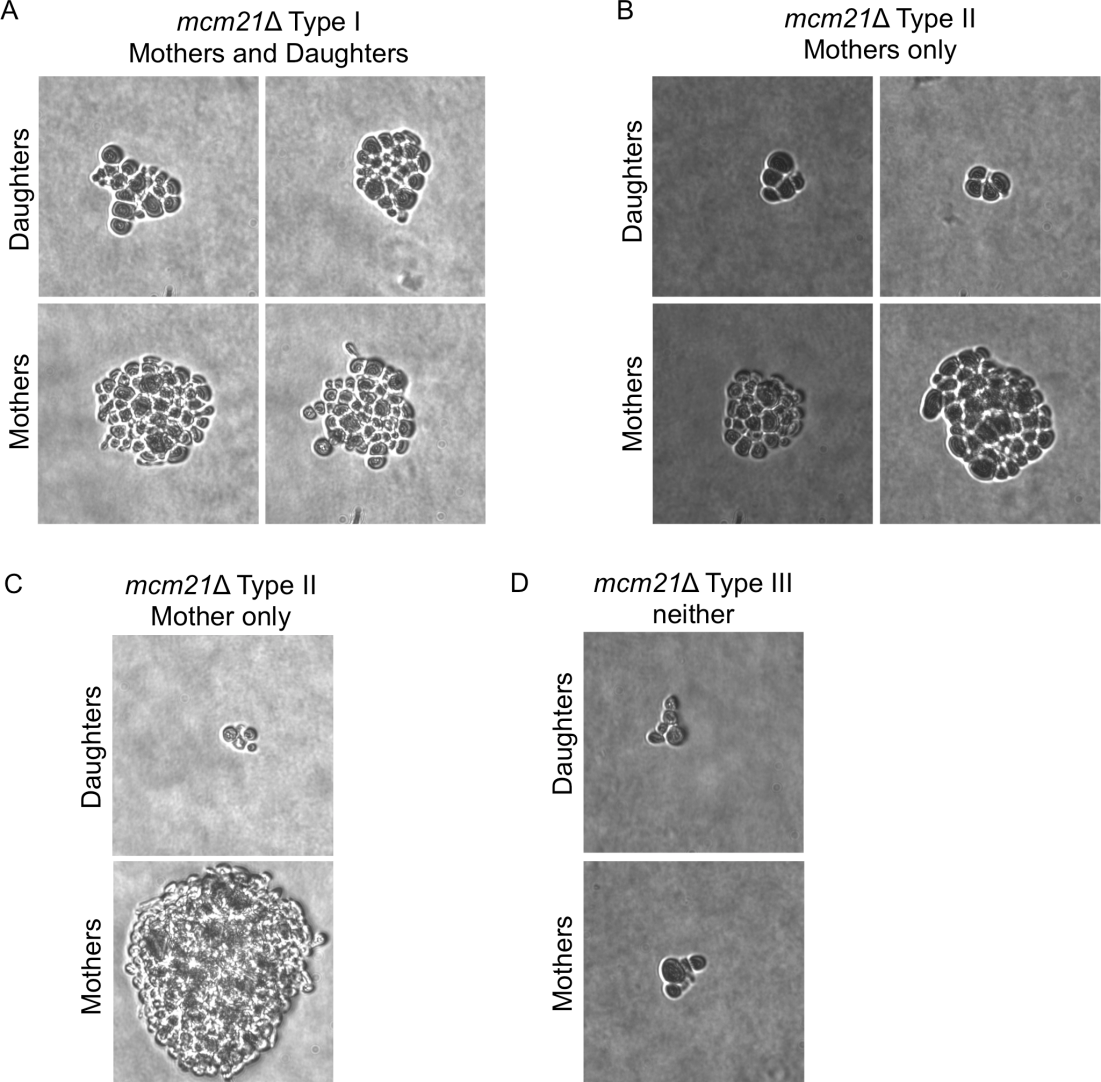
Pedigree analysis of *mcm21Δ*. G1 cells (*mcm21*Δ *GAL-CEN3*) were micromanipulated on YEP-galactose plates and after the first division mothers and daughters were separated and allowed to grow approximately 24 h. Images of microcolonies were photographed. A. Both mother and daughter cells divided multiple times. B. Only mother cells divided multiple times. C. Only mother cell divided and grew into an observable colony. D. Neither mother nor daughter progressed beyond 2–3 divisions.

The pedigree analysis of *mcm21*Δ cells containing the *GAL-CEN3* centromere revealed a complex effect on viability. In 14 of 70 (20%) cases, both mothers and daughters formed microcolonies of roughly 20 cells after 24 h (Figs 1C and 6A), not statistically different from the *GAL-CEN3* WT case. The majority of pedigrees (38/70, 54.3%) had a mother than gave rise to 10–20 cells after 24 h whereas the daughter was arrested after two cell divisions (usually 4 cells); this is indicative of Chr 3 mis-segregation where the daughter failed to receive the chromosome but can divide once (Figs 2C, 6B and 6C). There were also 18/70 (25.7%) instances in which neither mother nor daughter progressed beyond about a single division (Figs 2C and 6D). Surprisingly, although many mothers and some daughters divided multiple times, after 100 h of growth, the majority of these *mcm21*∆ microcolonies did not give rise to visible colonies, consistent with the reduced viability in the population measurements (22%). In the images shown, only the mother in Fig 6C grew into a visible colony. These results suggest that continuous expression of *GAL-CEN3* in *mcm21*Δ results in the failure of mother cells to produce viable daughters. The initial increase in cell number, (> 20 cells), appears to be the limited proliferation of daughter cells, produced, once each 2 h, lacking Chr 3. We conclude that depletion of pericentric cohesin diminishes the segregation capabilities of the *GAL-CEN3* chromosome on galactose, but the severity of the defect is manifest after several generations (Fig 1).

### Redistribution of cohesin to the pericentromere biases *GAL-CEN3* toward the active state

Cohesin functions in chromosome looping, barrier formation and pericentromere structure. The strategy of depleting a kinetochore component (Mcm21) to reduce cohesin function is compromised by potential kinetochore-specific roles for Mcm21 and the COMA complex. To test whether the cohesin concentration directly shifts *GAL-CEN3* toward a functional state, we utilized a strategy to increase pericentric cohesin in the absence of the loading factor Mcm21. Cohesin is recruited to the pericentromere via COMA (Ctf19, Okp1, Mcm21, Ame1 complex [29]) and to the rDNA via Sir2 [14,30]. Upon deletion of *MCM21* pericentromeric cohesin is decreased, while the concentration of cohesin increases in the rDNA [31]. Similarly, deletion of *sir2∆* results in decreased cohesin at the rDNA, and increased cohesin in the pericentromere [31]. As reported by Stephens *et al*., [31] we find a decrease in pericentric cohesin concentration in the absence of *mcm21∆* (~40% Fig 5B). In the *mcm21∆ sir2∆* double mutant the concentration of pericentric cohesin returns to wild-type levels (~1.67X increase, Fig 5B). Furthermore, pericentric cohesin in the *mcm21∆ sir2∆* double mutant is faithfully organized as evidenced by the barrel structure around the spindle (Fig 5C). Viability of cells containing the *GAL-CEN3* chromosome on galactose was restored to 80% in the double *mcm21∆ sir2∆* mutants, close to wild-type levels (Fig 1). Likewise, the distribution of the galactose-grown *GAL-CEN3* chromosome on or off the spindle in the double mutants was shifted toward the distribution observed in *GAL-CEN3* cells (3X increase of on axis separated spots in metaphase and 2X increase in segregated spots in late anaphase relative to *mcm21∆*, Fig 3C). Thus cohesin contributes to the fidelity of chromosome segregation in cells with transcriptionally compromised kinetochores (Figs 1 and 3C).

### Pericentric cohesin regulates the ability of *GAL-CEN* to recruit Mif2

**Fig 7:**
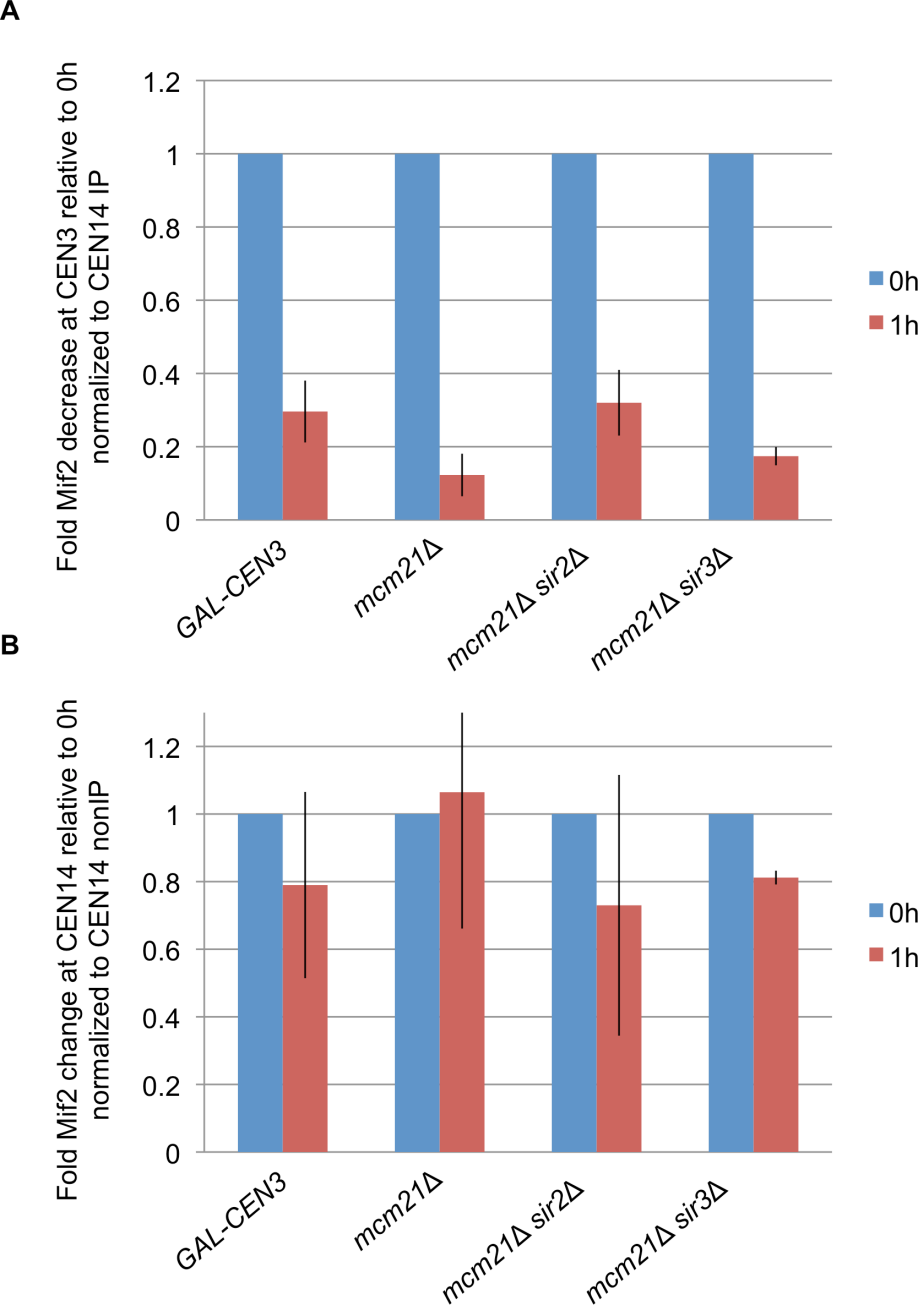
Deletion of *MCM21* leads to a greater reduction in Mif2 levels after activation of CEN3. A) Mif2 ChIP at *CEN3* normalized to *CEN14* IP. Mif2 fold change 1 hour after inactivation of *CEN3* by addition of galactose was measured in *GAL-CEN3* (SGD10.2), *mcm21Δ*, *mcm21Δ sir2Δ* and *mcm21Δ sir3Δ*. Mif2 levels in *mcm21Δ* are significantly lower than *GAL-CEN3* (p = 0.02) and *mcm21Δ sir2Δ* (p = 0.016) but not significantly different from *mcm21Δ sir3Δ*. B) Mif2 ChIP at *CEN14* normalized to *CEN14* nonIP. Mif2 fold change 1 hour after inactivation of *CEN3* by addition of galactose was measured in *GAL-CEN3* (SGD10.2), *mcm21Δ*, *mcm21Δ sir2Δ* and *mcm21Δ sir3Δ*.

To test whether pericentric cohesin directly modifies the kinetochore we used ChIP to assess the concentration of Mif2 at the *GAL-CEN3* locus. Mif2, the yeast ortholog of mammalian CENP-C, is a centromeric protein that localizes to the kinetochore and is required for spindle integrity during the metaphase to anaphase transition [32,33,34]. Mif2 levels at *GAL-CEN3* are reduced to 30% upon growth on galactose (Fig 7A), in agreement with previous findings [7]. Mif2 levels at *CEN14* are not significantly lower after galactose addition (Fig 7B), indicating that the reduction in Mif2 levels at *GAL-CEN3* is caused by the local transcriptional inactivation of *CEN3*.

Because deletion of *MCM21* leads to a reduction in viability and a defect in sister centromere separation after *CEN3* inactivation we hypothesized that deletion of *MCM21* would lead to a greater reduction in Mif2 levels after shift to galactose. Indeed, Mif2 levels decreased to 12% following *CEN3* inactivation (Fig 7A), reflecting the reduced ability of *GAL-CEN3* to direct chromosome segregation without Mcm21.

To address whether the reduction of Mif2 in *mcm21Δ* reflects the greater inhibition of centromere via transcriptional inactivation, we performed RT-qPCR to quantitate transcription of *GAL-CEN3* locus in the *GAL-CEN3* WT, *mcm21Δ* and *mcm21Δ sir2Δ* strains. Induction of the *GAL1* gene one hour following galactose induction was similar in *GAL-CEN3* WT, *mcm21Δ* and *mcm21Δ sir2Δ* when normalized to ACT1 (S4A Fig). *GAL-CEN3* transcription was not altered upon deletion of *mcm21Δ* (S4B Fig). We then tested the transcription level on the side of *CEN3* distal from the GAL promoter. While in *GAL-CEN3* WT cells transcript levels distal from the GAL promoter where reduced to approximately 0.6 of the transcript immediately adjacent to the GAL promoter, in *mcm21*Δ the transcript levels on both sides of *CEN3* were identical (S4C Fig). These results indicate that cohesin promotes proper segregation not by controlling transcription but by a different mechanism, such as ensuring proper kinetochore assembly. Accordingly, when cohesin recruitment is impaired the kinetochore does not pose a barrier for transcription.

As deletion of *SIR2* was shown to rescue the viability of *mcm21*Δ, we tested if deletion of *SIR2* would also rescue Mif2 levels in *mcm21*Δ. Strikingly, Mif2 levels 1 h following *CEN3* inactivation by galactose in *mcm21*Δ *sir2*Δ were 32% (Fig 7A) comparable to 30% in *GAL-CEN3* WT. Sir2’s recruitment of cohesin to the rDNA is independent of the Sir3 and Sir4 proteins that are required with Sir2 in silencing of telomeres and heterochromatic regions [35,36]. Sir2’s effect at *GAL-CEN3* could be through its gene silencing activity rather than its role at rDNA. To distinguish between these possibilities we tested whether deletion of *SIR3* would also rescue viability and Mif2 levels in *mcm21*Δ. Unlike the viability in *mcm21*Δ *sir2*Δ (80%), *mcm21*Δ *sir3*Δ exhibited 23% viability on galactose plates, comparable to 22% in *mcm21*Δ (Fig 1). Likewise, Mif2 levels were reduced to 17% in *mcm21*Δ *sir3*Δ, comparable to *mcm21*Δ (12% p = 0.11) but significantly different than *mcm21*Δ *sir2*Δ (32% p = 0.027). These results suggest that *SIR2* deletion rescues *mcm21*Δ by increasing cohesin recruitment to the pericentromeric region, and not through its silencing function.

### Direct tethering of cohesin promotes centromere separation

To directly assess the mechanistic role of pericentric cohesin in centromere function we introduced binding sites for LexA adjacent to *CEN3* and expressed a Sir2-LexA fusion protein [37]. Sir2 recruits cohesin and can promote sister chromatid cohesion [30]. Loss of Chr 3 in a *MAT*α haploid can be assayed by measuring the transient creation of **a**-like mating cells by their ability to mate with another *MAT*α strain [38]. The fidelity of segregation for Chr 3 containing the LacO/LexA repeat arrays was indistinguishable from Chr 3 lacking the foreign DNA (via quantitative mating assay, 1.34 x 10^−5^ vs. 0.7 x 10^−5^ with and without LexA-LacO arrays, respectively). Upon expression of the Sir2-LexA fusion protein, wild-type levels of segregation were maintained (3.66 x 10^−5^ vs. 1.34 x 10^−5^ with and without Sir2-LexA fusion protein, respectively). Thus, additional recruitment of cohesin to a centromere-proximal position does not further enhance chromosome segregation fidelity.

**Fig 8:**
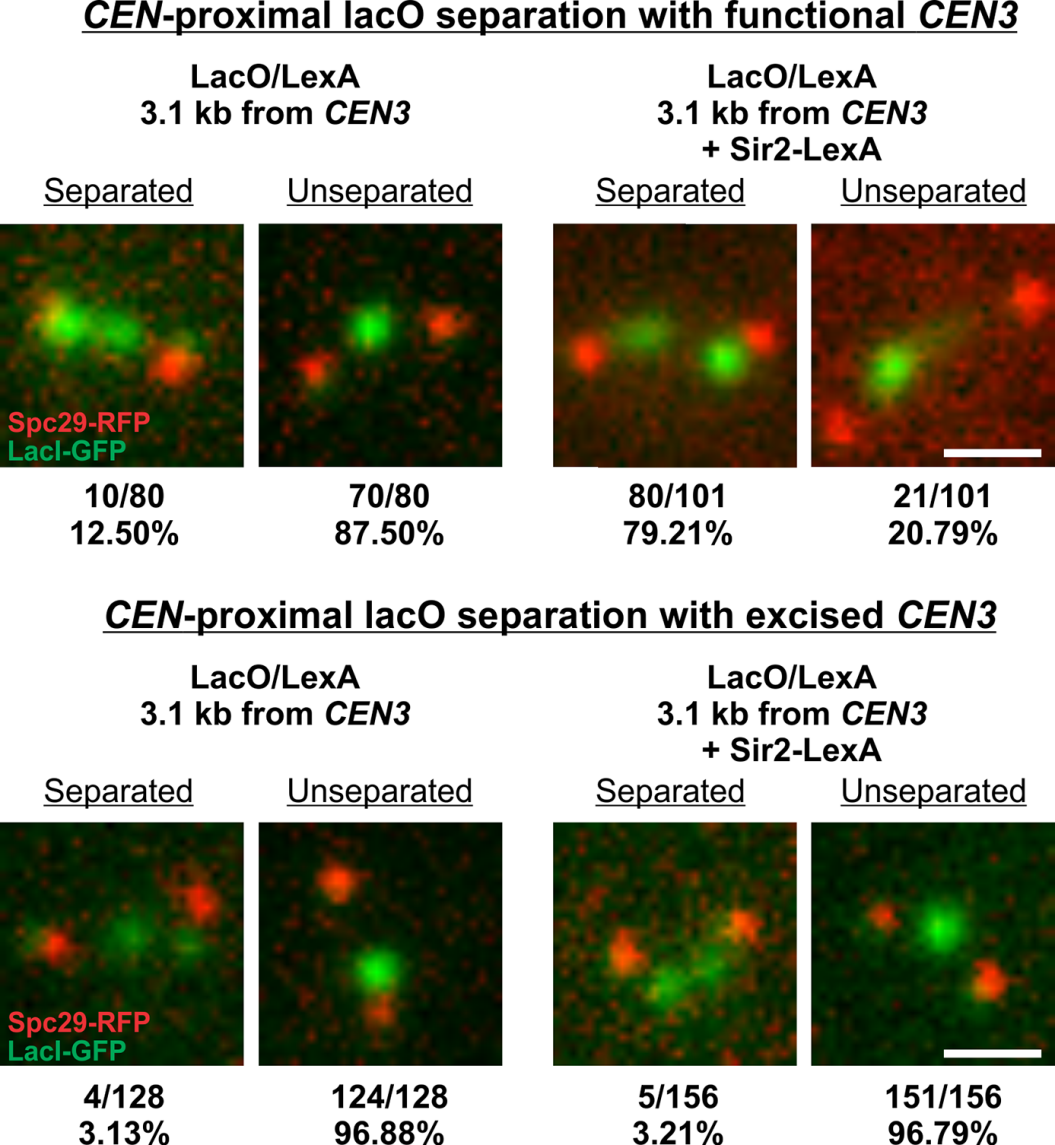
Pericentric sister chromatid separation in the presence or absence of tethered Sir2. The percent of separated sister chromatids in cells containing LexA/LacO binding sites proximal to the endogenous centromere on Chr 3 either with (right) or without (left) plasmid expressing Sir2-LexA fusion protein. Top panel: Chromosomes with intact *CEN3*. Bottom panel: Chromosomes with excised *CEN3*. Representative images (green lacI-GFP, red spindle poles Spc29-RFP) of cells in mitosis. Scale bar 1 micron.

The function of pericentric cohesin was revealed through live cell imaging of chromatin proximal to *CEN3*. Using centromere-proximal LacO-LacI GFP to visualize the pericentromere, we found about 12% of cells had separated or stretched sister centromeres in the absence of Sir2-LexA (Fig 8). Upon expression of a Sir2-LexA fusion protein, the fraction of separated sister centromeres increased dramatically to 79% (Fig 8). Thus, local recruitment of cohesin promotes additional centromere separation in metaphase.

### Redistribution of cohesin from the rDNA to the centromere restores the distribution of sister kinetochore clusters

**Fig 9:**
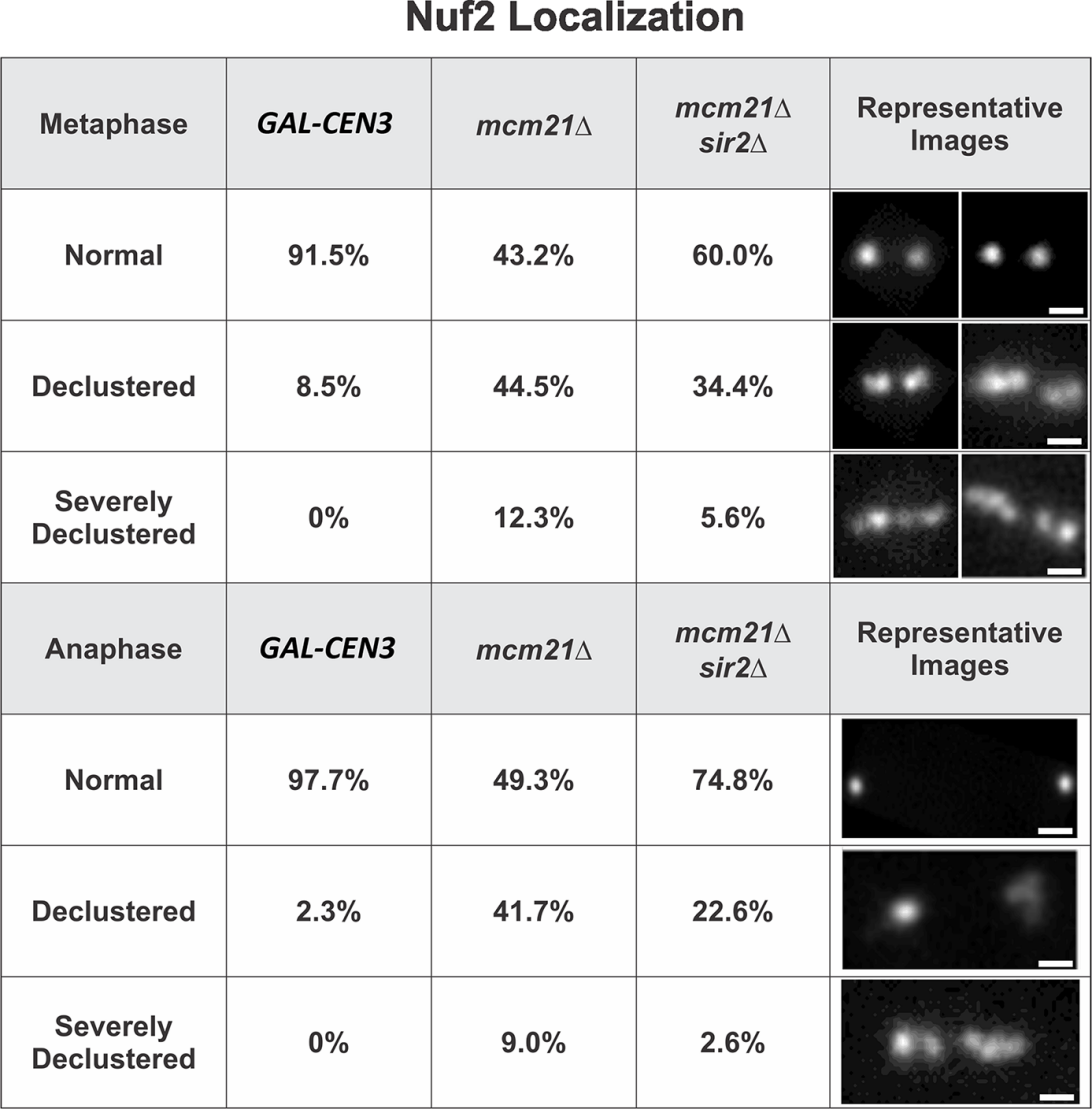
Loss of Sir2 suppresses kinetochore declustering in *mcm21Δ* mutants. The distribution of Nuf2 in *GAL-CEN3*, *mcm21*∆ and *mcm21∆ sir2∆* mutants. Nuf2-GFP (one of the 4 proteins in the Ndc80 complex) appears as two clusters of sister kinetochores in metaphase (Normal, *GAL-CEN3* 91.5%, top panel) and anaphase (Normal, *GAL-CEN3* 97.7% bottom panel). Nuf2 declusters into several spots along the spindle axis in the absence of *mcm21∆* (declustered and severely declustered *mcm21∆*) in metaphase and anaphase. The distribution of clustered and declustered Nuf2 in the double *mcm21∆ sir2∆* mutant (Nuf2 *mcm21∆ sir2∆*) is intermediate between *GAL-CEN3* WT and *mcm21∆* mutant. (Right) Representative images of normal clustered, declustered and severely declustered Nuf2-GFP in the kinetochore. For metaphase, *GAL-CEN3* n = 94, *mcm21Δ* n = 155, *mcm21Δ*, *sir2*Δ n = 125; anaphase, wt n = 86, *mcm21Δ* n = 134, *mcm21Δ*, *sir2Δ* n = 115.

Pericentric cohesin contributes to the clustering of 32 replicated kinetochores into two foci on the metaphase spindle [26]. If the loss of kinetochore clustering reflects the reduced concentration of cohesin, as opposed to another function of the COMA complex, then restoration of pericentric cohesin levels in the *sir2∆* mutant will also restore kinetochore clustering. To test this we examined kinetochore clustering in cells containing one of the components of the NDC80 outer kinetochore complex (Nuf2-GFP). In *GAL-CEN3* cells, more than 90% of cells exhibit two clusters of Nuf2 in both metaphase and anaphase (Fig 9). In the absence of *MCM21* only 43% of cells contain two focused clusters, while the remaining cells exhibit declustered kinetochores that are distributed throughout the metaphase spindle. 12.3% of the cells contained severely declustered kinetochores along the entire spindle axis (Fig 9). In anaphase, with spindles > 5 μm, the declustered phenotype persists in ~50% of cells (anaphase declustered and severely declustered, Fig 9). In *mcm21∆ sir2∆* double mutant kinetochore clustering is partially restored. In metaphase, the clustered phenotype is increased from 43% in *mcm21∆* to 60% in *mcm21∆ sir2∆*. Likewise, the severely declustered phenotype decreased from 12.5% to 5.6%. In anaphase the clustered phenotype is observed in 75% of cells, up from 50% in *mcm21∆* mutants (Fig 8). The redistribution of cohesin to the pericentromere contributes to kinetochore positioning as well as kinetochore protein recruitment (MIF2) and segregation fidelity of the *GAL-CEN3* on galactose.

## Discussion

Point centromeres can be conditionally inactivated upon induction of a proximal transcriptional promoter. The ability to regulate chromosome segregation through this construct has been a powerful tool in many studies of chromosome stability and aneuploidy [6,39,40]. By placing the conditional centromere as the sole site for kinetochore assembly in one chromosome we have found that chromosome segregation fidelity is reduced upon transcriptional inactivation of the centromere, but not abolished.

Transcription does not completely remove kinetochore proteins. The remaining components assemble into a functional kinetochore with sufficient time and accuracy to allow cell growth into a colony. The residual function is evident compared to the complete removal of the centromere via DNA excision, which drops viability to less than 1%. In this study, we demonstrate we demonstrate that pericentromeric cohesion modulates the deleterious effects of *GAL-CEN* transcription and allows for greater retention of the Mif2 kinetochore protein. Cohesin can be redistributed to the pericentromere in *sir2∆* mutants, indicative of a dynamic pool that equilibrates between the two major sites of cohesin binding (nucleolus and pericentromere) [30,31]. Cohesin is uniformly distributed around the spindle in metaphase and is physically stable over several minutes [15,41,42]. The redistribution from one pool (rDNA) to the other (pericentromere) most likely reflects the redirection of cohesin to sites of loading as a consequence of the increase in available protein. Upon shifting the equilibrium of cohesin to the pericentromere in *mcm21*∆ *sir2∆* double mutants, viability in cells with a *GAL-CEN3* chromosome returns to wild-type levels. Furthermore, kinetochore protein concentration returns to levels observed at *GAL-CEN3* in WT cells. Sir2 does not silence the *GAL-CEN*, as deletion of *SIR3* does not alter cell viability or kinetochore protein levels.

**Fig 10:**
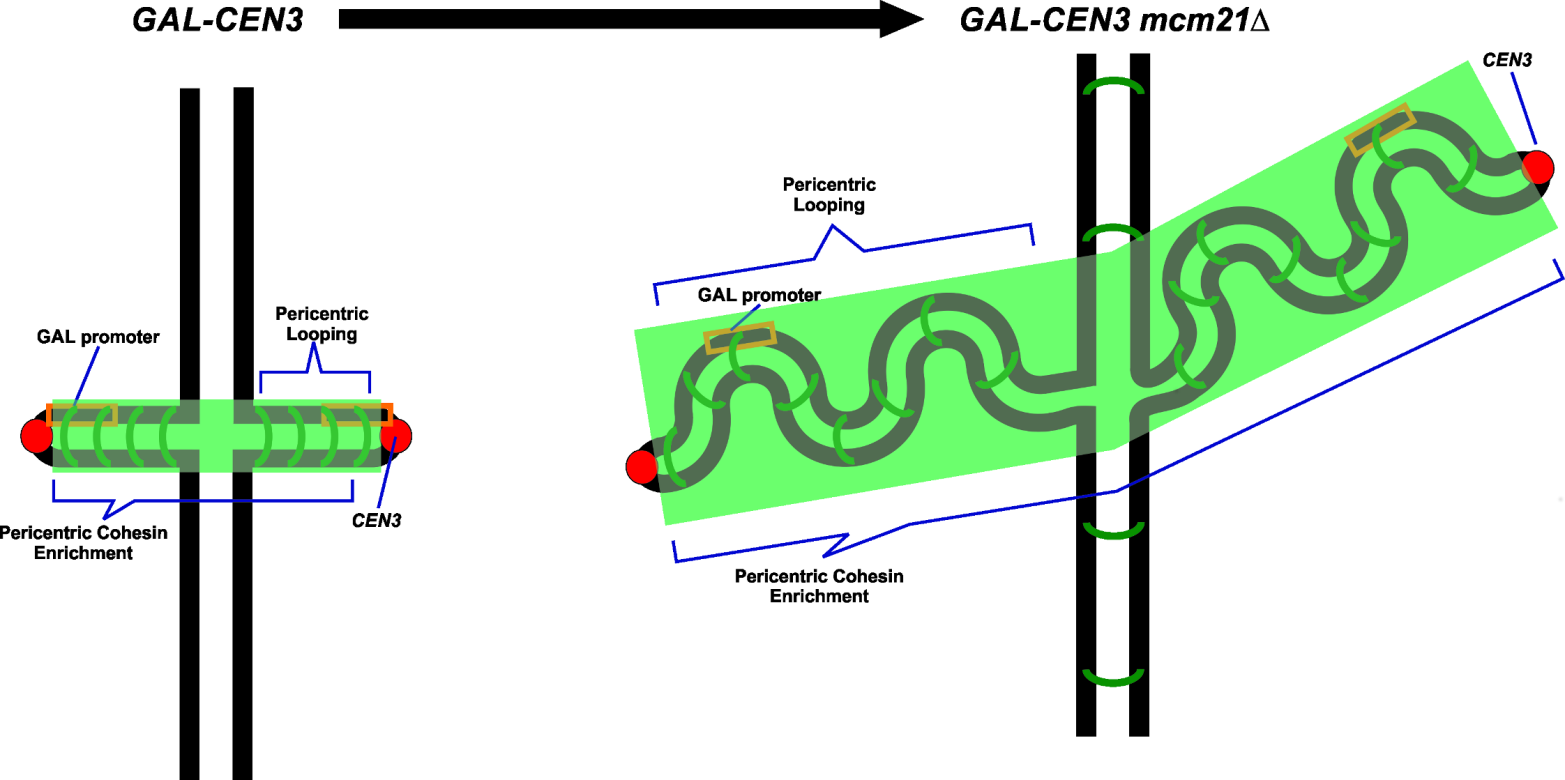
A model for cohesin contribution to pericentric conformation and proper segregation. Cohesin stiffens the pericentric region in metaphase. In the situation where the centromere is inactivated by a proximal promoter, cohesin-dependent stiffening is sufficient for the centromere to dictate kinetochore assembly and allow for viable segregation. In *mcm21Δ* the defect in cohesin recruitment results in a defective pericentric structure leading to segregation defects resulting in lower viability.

The role of cohesin in the pericentromere remains enigmatic. Cohesin is not holding sister chromatids together, as they are separated by 400–800 nm in metaphase. This study suggests that cohesin contributes to the conformation of pericentric chromatin that is favorable for kinetochore assembly (Fig 10). It is unlikely that cohesin directly recruits kinetochore proteins as there are no direct interactions, and in vivo the pericentric cohesin barrel is well separated from the kinetochore/microtubule attachment complex. It has been suggested that proteins such as Sgo1 contribute to the bias that favors sister centromeres to face opposite poles [24,43]. The barrel of pericentric cohesin could be the physical manifestation of such a mechanism. By assembling cohesin between sister centromeres, the centromeres will be inherently pushed apart and thereby favoring the centromere to lie on the surface of the chromosome. In this scenario, the recruitment of Mif2 via cohesin reflects the geometric configuration in the presence of cohesin.

Cohesin may also function in stiffening chromatin. We have recently found that DNA loops in the pericentromere generate intracentromere tension in mitosis [44]. This is due to the proximity of the radial loops and the thermodynamics consequences along the axis of DNA coincident with the spindle microtubules. DNA loops restrict the axial DNA from adopting a random coil, instead they generate an axial force. The centromeres lie at the apex of this axial DNA and are therefore physical extruded to the surface of the chromosome. In addition, we have found that increasing the number of cohesin ring molecules around a circular plasmid decreases the ability of the plasmid to collapse into a random coil. Instead, cohesin stiffens the plasmid as evidenced by the increase in radius of the plasmid at thermodynamic equilibrium. Ring-like proteins at sufficient density can stiffen chromatin, providing a mechanism for shaping chromatin structure *in vivo*. This points to a novel function for cohesin ring complexes that may have significant biological implications, particularly at the centromere.

The role of cohesin in confinement and segregation is reminiscent of the role of condensin in compaction and segregation of the bacterial nucleiod [45,46]. The timing of condensin-mediated compaction of DNA in bacteria is closely linked to the chromosome segregation cycle and cell division. In eukaryotes, condensin and cohesin loading is coupled to DNA replication [12,47] and chromosomes are condensed well before anaphase chromosome segregation. Linking chromosome compaction with segregation in bacteria may reflect a strategy to convert cohesin and/or condensin-mediated compaction forces into mechanisms that promote strand separation [48,49]. These compacting proteins push DNA out of thermal equilibrium and upon protein release (e.g. separase cleavage of cohesin) the DNA will naturally expand. In a confined space such as *E*. *coli*, this energy can result in the physical segregration of two molecules [50]. In eukaryotes, pericentric cohesin may play a similar role. The enrichment of cohesin in centromeric chromatin may be indicative of stiffening and confinement functions of these conserved proteins.

## Materials and Methods

### Strain construction

Strain SGD10.2 was constructed using plasmid JC313 *GAL-CEN3* [51]. JC313 *GAL-CEN3* was digested with EcoRI to create the transformation fragment. The fragment was used to transform strain KBY8039. Strain list in Table 1.

**Table 1:** Strain list.

Strain	Genotype
473**a**	MAT**a** ade2 ura3 leu2 trp1 his3 can1-100
SGD10.2	MAT**a** ade2 ura3 leu2 trp1 his3 can1-100 LacINLSGFP:HIS3 LacO::URA3(at 3.8kb from CEN3, 10kb array) UraΔ::Nat GALCEN3::URA (JC313) Spc29-RFP:Hb
BNE2001	MAT**a** ade2 ura3 leu2 trp1 his3 can1-100 LacINLSGFP:HIS3 LacO::URA3(at 3.8kb from CEN3, 10kb array) UraΔ:Nat GALCEN3::URA (JC313) mcm21Δ::TRP Spc29-RFP::Hb
MT206	MAT**a** ade2 ura3 leu2 trp1 his3 can1-100 LacINLSGFP:HIS3 LacO::URA3(at 3.8kb from CEN3, 10kb array) UraΔ:Nat GALCEN3::URA (JC313) mcm21Δ::TRP Sir3Δ::KAN Spc29-RFP:Hb
KBY8176	MAT**a** ade2 ura3 leu2 trp1 his3 can1-100 LacINLSGFP:HIS3 LacO::URA3(at 3.8kb from CEN3, 10kb array) UraΔ::Nat GALCEN3::URA (JC313) sir2Δ::Kan Spc29-RFP::Hb
KBY8175	MAT**a** ade2 ura3 leu2 trp1 his3 can1-100 LacINLSGFP:HIS3 LacO::URA3(at 3.8kb from CEN3, 10kb array) UraΔ::Nat GALCEN3::URA (JC313) mcm21Δ::TRP sir2Δ::Kan Spc29-RFP::Hb
KBY1894	MAT**a** trp1Δ63 leu2Δ ura3-52 his3Δ200 lys2-8Δ1 Smc3-GFP::URA
KBY 9065	MATa trp1ΔD63 leu2Δ ura3-52 his3Δ200 lys2-8Δ1 Smc3-GFP::URA mcm21Δ::Nat Spc29-RFP::Hb
KBY 9152	MATa trp1Δ63 leu2Δ ura3-52 his3Δ200 lys2-8Δ1 Smc3-GFP::URA mcm21Δ::Nat sir2Δ::Kan Spc29-RFP::Hb
W3616-3C	MAT**a** CEN2∷pGal1-CEN2-URA3^Kl^ ade2-1 can1-100 his3-11,15 leu2-3,112 lys2 met17 trp1-1 ura3-1 RAD5
W3616-3A	MATα CEN2∷pGal1-CEN2-URA3^Kl^ ade2-1 can1-100 his3-11,15 leu2-3,112 lys2 met17 trp1-1 ura3-1 RAD5
DY6280	MAT**a** CEN3∷pGal1-CEN3-URA3^Kl^ ade2-1 can1-100 his3-11,15 leu2-3,112 lys2 met17 trp1-1 ura3-1 rad5-535
DY6296	MATα CEN3∷pGal1-CEN3-URA3^Kl^ ade2-1 can1-100 his3-11,15 leu2-3,112 lys2 met17 trp1-1 ura3-1 rad5-535
DY6282	MAT**a** CEN4∷pGal1-CEN4-URA3^Kl^ ade2-1 can1-100 his3-11,15 leu2-3,112 lys2 met17 trp1-1 ura3-1 rad5-535
DY6298	MATα CEN4∷pGal1-CEN4-URA3^Kl^ ade2-1 can1-100 his3-11,15 leu2-3,112 lys2 met17 trp1-1 ura3-1 rad5-535
DY6283	MAT**a** CEN5∷pGal1-CEN5-URA3^Kl^ ade2-1 can1-100 his3-11,15 leu2-3,112 lys2 met17 trp1-1 ura3-1 rad5-535
DY6299	MATα CEN5∷pGal1-CEN5-URA3^Kl^ ade2-1 can1-100 his3-11,15 leu2-3,112 lys2 met17 trp1-1 ura3-1 rad5-535
YFD0960	MATa (HOcs Deleted) hmlΔ::ADE1 hmrΔ::ADE1 ade1-100 leu2-3,112 lys5 trp1::hisG' ura3-52 ade3::GAL::HO Cen3HOcs::HPH, pFD025 (URA3+) inserted right of Cen3
KBY 8198.1	(YFD0960 MAT**a** (HOcs Deleted) hmlΔ::ADE1 hmrΔ::ADE1 ade1-100 leu2-3,112 lys5 trp1::hisG' ura3-52 ade3::GAL::HO Cen3HOcs::HPH, pFD025 (URA3+) inserted right of Cen3 mcm21Δ::TRP
KBY 8200.1	(YFD0960 MAT**a** (HOcs Deleted) hmlΔ::ADE1 hmrΔ::ADE1 ade1-100 leu2-3,112 lys5 trp1::hisG' ura3-52 ade3::GAL::HO Cen3HOcs::HPH, pFD025 (URA3+) inserted right of Cen3 mcm21Δ::TRP sir2Δ::KAN
KBY8213	(KBY8212.12 YFD0960 MAT**a** (HOcs Deleted) hmlΔ::ADE1 hmrΔ::ADE1 ade1-100 leu2-3,112 lys5 trp1::hisG' ura3-52 ade3::GAL::HO Cen3HOcs::HPH, pFD025 (URA3+) inserted right of Cen3 Gasser-NAT-target site inserted 3.1kb downstream of Cen3) pSR12 (lacO/lexA::LEU2)
KBY8216.3	(8213 (KBY8212.12 YFD0960 MAT**a** (HOcs Deleted) hmlΔ::ADE1 hmrΔ::ADE1 ade1-100 leu2-3,112 lys5 trp1::hisG' ura3-52 ade3::GAL::HO Cen3HOcs::HPH, pFD025 (URA3+) inserted right of Cen3 Gasser-NAT-target site inserted 3.1kb downstream of Cen3) pSR12 (lacO/lexA: LEU2 inserted at NAT)) His3p:LacI-GFP::NAT (pLKL58Y cut with AhdI and BspeI)
KBY8218.1	(KBY8213 YFD0960 MAT**a** (HOcs Deleted) hmlΔ::ADE1 hmrΔ::ADE1 ade1-100 leu2-3,112 lys5 trp1::hisG' ura3-52 ade3::GAL::HO Cen3HOcs::HPH, pFD025 (URA3+) inserted right of Cen3 Gasser-NAT-target site inserted 3.1kb downstream of Cen3) pSR12 (lacO/lexA::LEU2 inserted at NAT) pCSW1 (lexA-Sir2_243-562_ HIS3 Cen plasmid)
KBY8230 diploid	(YFD0960 MAT**a** ((HOcs Deleted) hmlΔ::ADE1 hmrΔ::ADE1 ade1-100 leu2-3,112 lys5 trp1::hisG' ura3-52 ade3::GAL::HO Cen3HOcs::HPH, pFD025 (URA3+) inserted right of Cen3) x YEF473α (trp1Δ63 leu2Δ ura3-52 his3Δ200 lys2-8Δ1))
KBY8231 diploid	(KBY8213 (YFD0960 MAT**a** ((HOcs Deleted) hmlΔ::ADE1 hmrΔ::ADE1 ade1-100 leu2-3,112 lys5 trp1::hisG' ura3-52 ade3::GAL::HO Cen3HOcs::HPH, pFD025 (URA3+) inserted right of Cen3) Gasser-NAT-target site inserted 3.1kb downstream of Cen3) pSR12 (lacO/lexA::LEU2)) x YEF473α (trp1Δ63 leu2Δ ura3-52 his3Δ200 lys2-8Δ1))
KBY8232 diploid	(KBY8218 (YFD0960 MAT**a** ((HOcs Deleted) hmlΔ::ADE1 hmrΔ::ADE1 ade1-100 leu2-3,112 lys5 trp1::hisG' ura3-52 ade3::GAL::HO Cen3HOcs::HPH, pFD025 (URA3+) inserted right of Cen3) Gasser-NAT-target site inserted 3.1kb downstream of Cen3) pSR12 (lacO/lexA::LEU2) pCSW1 (lexA-Sir2243-562 HIS3 Cen plasmid)) x YEF473 α (trp1Δ63 leu2Δ ura3-52 his3Δ200 lys2-8Δ1))

### Growth conditions

Plating assays were conducted using W303 and SGD10.2 cultures grown overnight in YP+Glucose liquid media. Serial dilutions were created and cells were plated onto YP+Glucose and YP+Galactose plates. Plates were incubated for 5–6 days at 25°C. To induce lacI-GFP for imaging, SGD10.2 cells were maintained on synthetic–HIS media. To inhibit centromere function of the *GAL-CEN3*, SGD10.2 was grown overnight in 5 ml of synthetic–HIS+glucose liquid media at 25°C. 50 μl of this culture was then transferred to 5 ml of synthetic–HIS +lactose liquid media and grown overnight at 25°C. On the day of imaging, 500 μl of 20% galactose was added to the SGD10.2 culture. After 3–4 hours of shaking at 25°C, cells were imaged.

### Imaging conditions

Images of plates were taken using a Canon CanoScan 4400F Scanner. Population imaging was performed on live cells immersed in rich, synthetic imaging media supplemented with 2% glucose or galactose. Time lapse, live-cell imaging was performed using cells immobilized on 25% gelatin slabs containing 2% Glucose or Galactose. Image acquisition was carried out using a Nikon Eclipse TE2000-U inverted microscope stand (Tokyo, Japan) with a 100X, 1.4 N.A. differential interference contrast (DIC) oil-immersion lens. Images were acquired with a Hammamatsu ORCA-ER CCD camera (Bridgewater, NJ). MetaMorph 7 software (Molecular Devices, Downington, Pennsylvania) controlled the microscope. Population imaging was performed using an acquisition protocol taking 5 fluorescence images at 0.5 μm axial steps and a single DIC image corresponding to the central fluorescence image. Exposure times ranged from 300–400 ms. For time lapse imagine, the same 5 step protocol was used at 2 minute intervals.

### Image analysis and creation

Distances were measured using the Measure Pixel tool in MetaMorph 7 software. To correct for random errors, each frame stack analysis was repeated three times. Data sets were exported into Microsoft Excel (Microsoft, Richmond, Washington) for analysis. Rates of *GAL-CEN3* movement were calculated by fitting a regression line to plots. *GAL-CEN3* to pole movement was defined as at least 3 consecutive time points of decreasing distance between the LacO/LacI-GFP focus and the SPC29-RFP focus. Slopes of regression lines were used to determine rates of movment. All models and schematics were created using CorelDRAW 11 software. Quantitation of Smc3-GFP fluorescence was performed as previously described [15]. A 16-pixel ×12-pixel rectangle (1040 nm ×780 nm) was manually placed around the Smc3-GFP signal between the spindle poles of metaphase cells with both spindle pole bodies (Spc29-RFP) in focus in the same z-plane. Background measured in a nuclear region away from the spindle axis was subtracted from the integrated value of Smc3-GFP fluorescence.

### Quantitative mating

Haploid cells with and without the LacO/LexA binding sites adjacent to *CEN3* were mated and diploids were selected. The diploids were mated to tester strains (KBY7523A) and plated onto selective media where only cells that have lost Chr 3 (due to chromosome loss, recombination or gene conversion) and consequently gained the ability to mate with the tester, were able to grow. Cells with LacO/LexA were transformed with pCSW1 (Sir2-LexA) and examined for quantitative mating in the same fashion [52,53].

### ChIP

Chromatin-immunoprecipitation was done as previously described [54]. Mif2 antibody was a generous gift of Doug Koshland.

### RT-qPCR

RNA was extracted using epicentre MasterPure Yeast RNA Purification kit. RNA was reverse transcribed using Thermo Fisher SuperScript IV with random hexamers. The resulting cDNA was analyzed by qPCR. GAL1 and GAL-CEN3 were normalized to ACT1 transcript.

## Supporting Information

S1 Fig As described in Fig 2 individual G1 cells were micromanipulated into an array on a YEPD plate and were monitored microscopically. Segregation of the *GAL-CEN* chromosome to the daughter was more successful when we monitored cells that were resuspended from streaks growing on plates than from liquid-grown cultures. When a cell had completed budding and a new bud just appeared one of the cells (presumably the slightly larger, mother cell), the mother and daughter cells were separated by micromanipulation and then observed approximately 12 hrs later to determine if the cell had grown into a microcolony of >20 cells or had arrested either as a single dumbbell or as a microcolony of <8 cells. For *GAL-CEN2* n = 32; *GAL-CEN3* n = 31; *GAL-CEN4* n = 24; *GAL-CEN5* n = 30. Type I: Mother viable, Daughter viable; Type II: Mother viable, Daughter dead; Type III: Mother dead, Daughter viable; Type IV: Mother dead, Daughter dead.
(TIF)

S2 Fig **A. Schematic of the *HOcut-CEN3* chromosome.** The chromosome contains an HO cut site (yellow) adjacent to *CEN3* on Chr 3, flanked by two regions of homology (orange) [22]. A lacO/LexA array was integrated 3.1 kilobases from the centromere sequence of the *HOcut-CEN3* chromosome. The centroid of the LacO array is 8.1kb from *CEN3*. Thick black lines represent chromosome arms. The chromosome is drawn based upon direct observations in live cells. The centromeres (red) are separated by approximately 800 nm. Cohesin (green) is enriched in the pericentromere region, about 50 kb surrounding each centromere. Upon induction of HO (on galactose carbon source) the repair via homologous sequences (orange) result in a complete deletion of the centromere (Post Cut). B. Viability was derived from the percentage of colony forming units on galactose versus glucose. From the left are wildtype HO-*CEN3*, HO-*CEN3 mcm21Δ* andHO-*CEN3 mcm21Δ sir2Δ* mutants (Gal).
(TIF)

S3 Fig Sister chromosome arm separation in wild type and *mcm21Δ* cells. Chromosome arm separation was monitored via introduction of LacO array 240 kb from the centromere on chromosome 2. The fraction of one vs. two spots in single cells was determined.
(TIF)

S4 Fig The effect of galactose induction on transcript levels of *GAL1* and *GAL-CEN3*. Transcript of *GAL1* and *GAL-CEN3* 1 h after galactose induction were normalized to *ACT1* transcript levels. A) *GAL1* transcript 1 h after galactose induction was approximately 5 fold higher than *ACT1* levels. The abundance of *GAL1* transcript was similar to *GAL-CEN3* in *mcm21*Δ and *mcm21*Δ *sir2*Δ. B) *GAL-CEN3* transcript 1 h after galactose induction was approximately 0.7 of *ACT1* levels. *GAL-CEN3* transcript in *mcm21Δ* was 0.5 of *ACT1*. This difference from *GAL-CEN3* was not statistically significant (p **=** 0.31). In *mcm21*Δ *sir2*Δ the transcript was 0.4 relative to *ACT1*. The difference from *GAL-CEN3* was not statistically significant (p **=** 0.08). The difference from mcm21Δ was not statistically significant (p **=** 0.6). C) Ratio of the *GAL-CEN3* transcript on both sides of the centromere. Transcript levels 300 bp after *CEN3* (distal) were divided by transcript levels between the GAL1-10 promoter (proximal) and normalized to *ACT1*.
(TIF)
