# Supplementary figures and images for: A Cohesin-Based Partitioning Mechanism Revealed upon Transcriptional Inactivation of Centromere
Source: PLoS Genet. 2016 Apr 29;12(4):e1006021. doi: 10.1371/journal.pgen.1006021 (PMC4851351; doi:10.1371/journal.pgen.1006021)

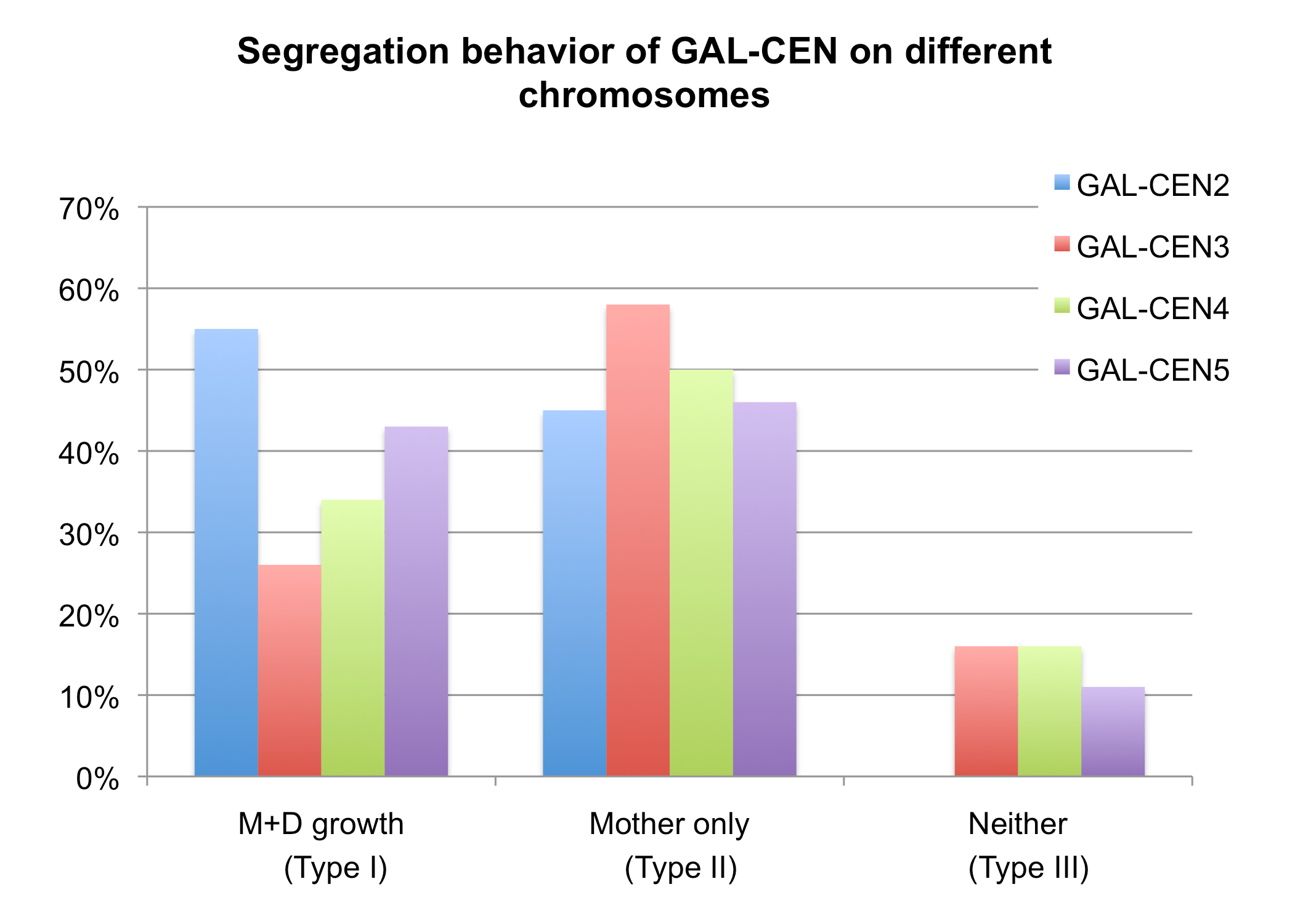

Supplement: S1 Fig — Segregation of the GAL-CEN chromosome to the daughter was more successful when we monitored cells that were resuspended from streaks growing on plates than from liquid-grown cultures. When a cell had completed budding and a new bud just appeared one of the cells (presumably the slightly larger, mother cell), the mother and daughter cells were separated by micromanipulation and then observed approximately 12 hrs later to determine if the cell had grown into a microcolony of >20 cells or had arrested either as a single dumbbell or as a microcolony of <8 cells. For GAL-CEN2 n = 32; GAL-CEN3 n = 31; GAL-CEN4 n = 24; GAL-CEN5 n = 30. Type I: Mother viable, Daughter viable; Type II: Mother viable, Daughter dead; Type III: Mother dead, Daughter viable; Type IV: Mother dead, Daughter dead. (TIF) [file pgen.1006021.s001.tif]

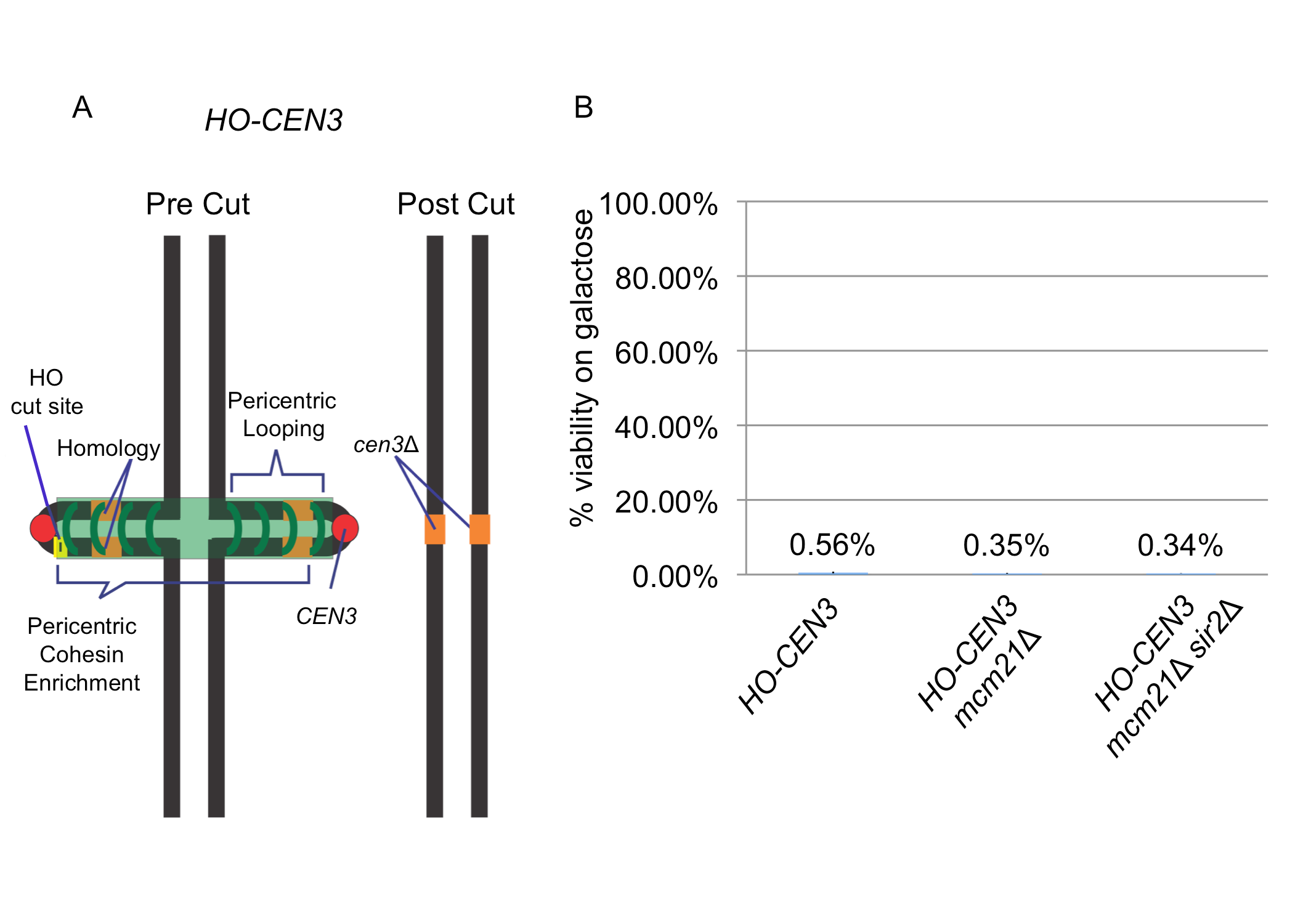

Supplement: S2 Fig — A. Schematic of the HOcut-CEN3 chromosome. The chromosome contains an HO cut site (yellow) adjacent to CEN3 on Chr 3, flanked by two regions of homology (orange) [22]. A lacO/LexA array was integrated 3.1 kilobases from the centromere sequence of the HOcut-CEN3 chromosome. The centroid of the LacO array is 8.1kb from CEN3. Thick black lines represent chromosome arms. The chromosome is drawn based upon direct observations in live cells. The centromeres (red) are separated by approximately 800 nm. Cohesin (green) is enriched in the pericentromere region, about 50 kb surrounding each centromere. Upon induction of HO (on galactose carbon source) the repair via homologous sequences (orange) result in a complete deletion of the centromere (Post Cut). B. Viability was derived from the percentage of colony forming units on galactose versus glucose. From the left are wildtype HO-CEN3, HO-CEN3 mcm21Δ andHO-CEN3 mcm21Δ sir2Δ mutants (Gal). (TIF) [file pgen.1006021.s002.tif]

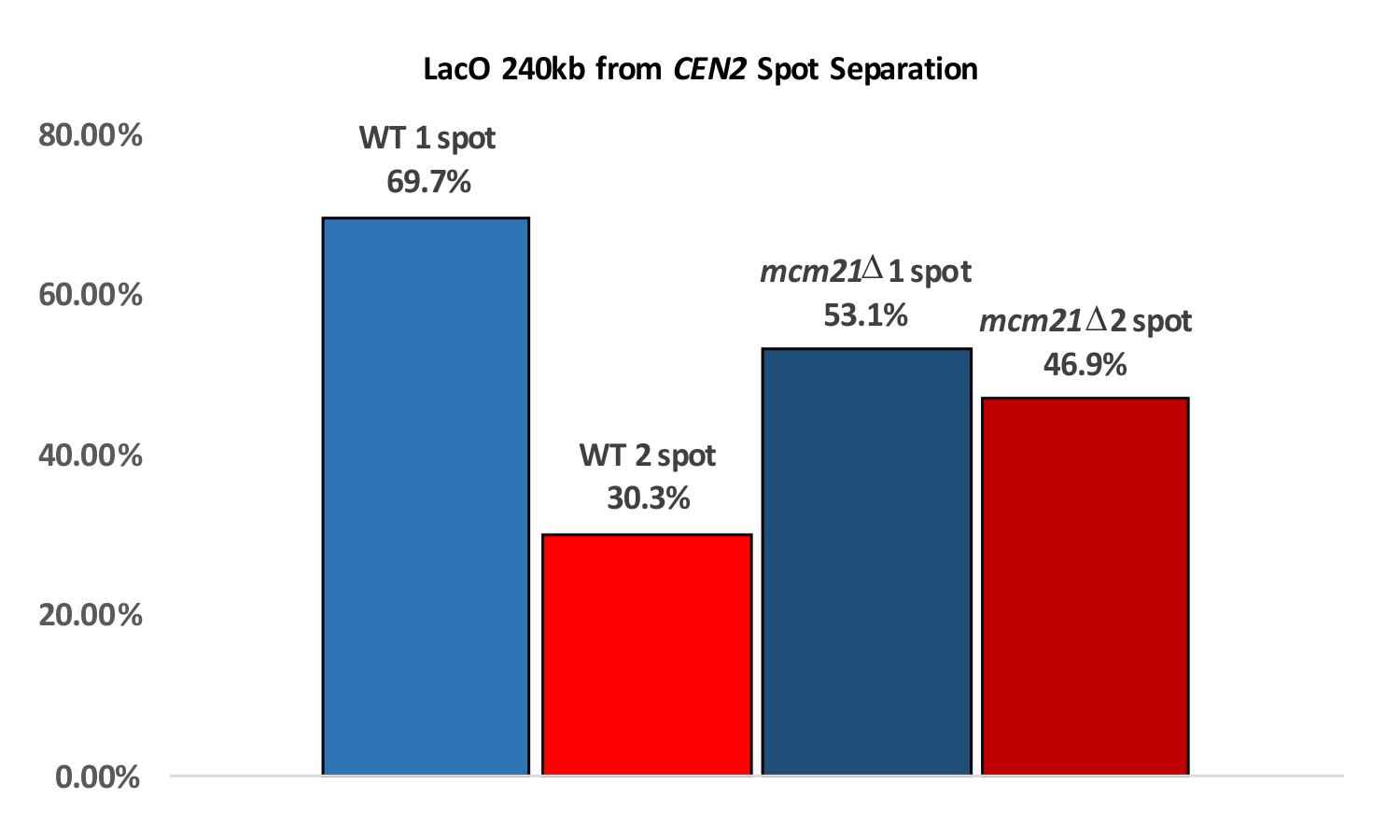

Supplement: S3 Fig — Chromosome arm separation was monitored via introduction of LacO array 240 kb from the centromere on chromosome 2. The fraction of one vs. two spots in single cells was determined. (TIF) [file pgen.1006021.s003.tif]

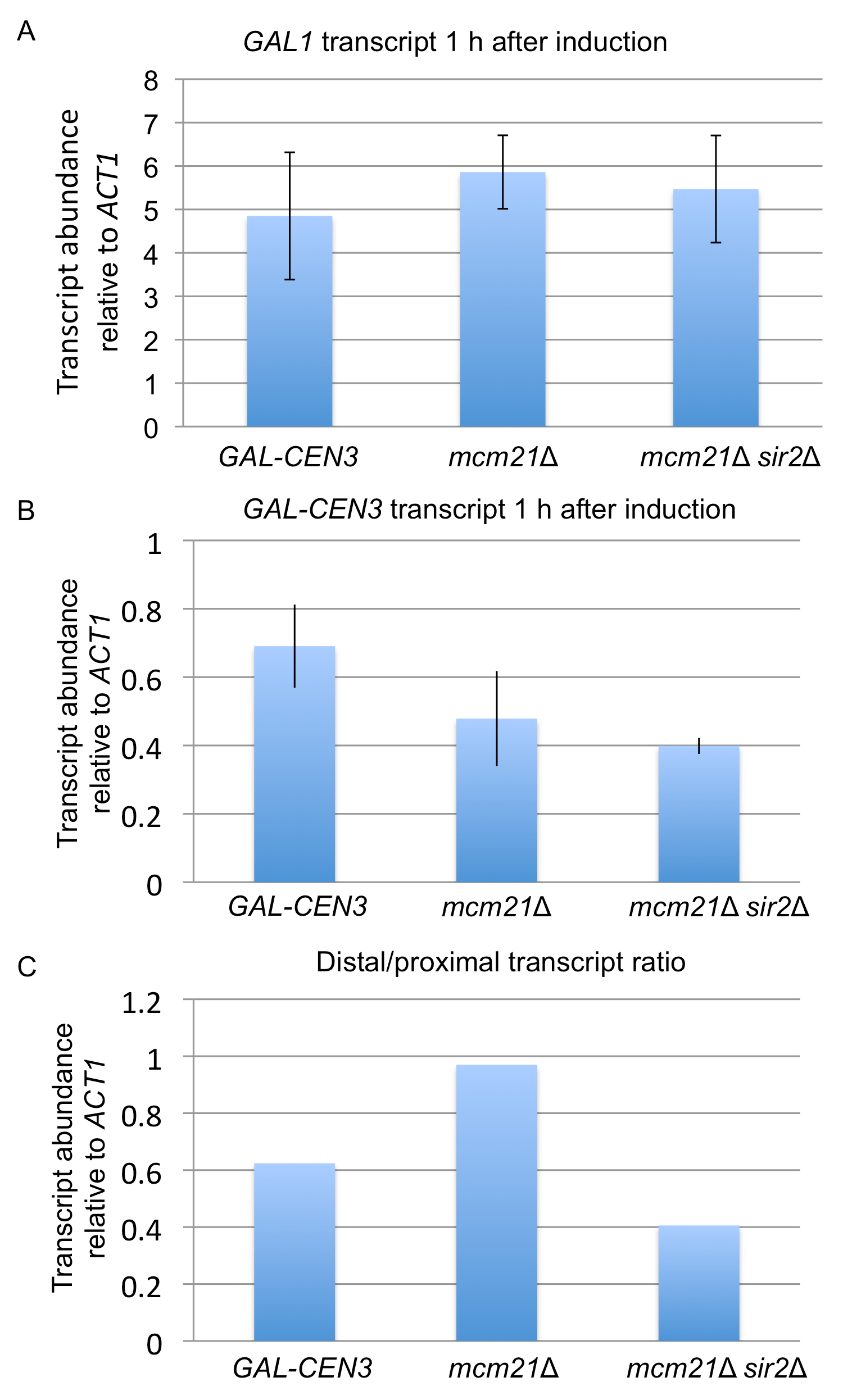

Supplement: S4 Fig — Transcript of GAL1 and GAL-CEN3 1 h after galactose induction were normalized to ACT1 transcript levels. A) GAL1 transcript 1 h after galactose induction was approximately 5 fold higher than ACT1 levels. The abundance of GAL1 transcript was similar to GAL-CEN3 in mcm21Δ and mcm21Δ sir2Δ. B) GAL-CEN3 transcript 1 h after galactose induction was approximately 0.7 of ACT1 levels. GAL-CEN3 transcript in mcm21Δ was 0.5 of ACT1. This difference from GAL-CEN3 was not statistically significant (p = 0.31). In mcm21Δ sir2Δ the transcript was 0.4 relative to ACT1. The difference from GAL-CEN3 was not statistically significant (p = 0.08). The difference from mcm21Δ was not statistically significant (p = 0.6). C) Ratio of the GAL-CEN3 transcript on both sides of the centromere. Transcript levels 300 bp after CEN3 (distal) were divided by transcript levels between the GAL1-10 promoter (proximal) and normalized to ACT1. (TIF) [file pgen.1006021.s004.tif]
